# *Stenotrophomonas maltophilia* promotes lung adenocarcinoma progression by upregulating histone deacetylase 5

**DOI:** 10.3389/fmicb.2023.1121863

**Published:** 2023-02-01

**Authors:** Jiyu Shen, Yalan Ni, Qijie Guan, Rui Li, Hong Cao, Yan Geng, Qingjun You

**Affiliations:** ^1^Department of Oncology, Affiliated Hospital of Jiangnan University, Wuxi, China; ^2^Key Laboratory of Carbohydrate Chemistry and Biotechnology, Ministry of Education; School of Biotechnology, Jiangnan University, Wuxi, Jiangsu, China; ^3^National Engineering Research Center of Cereal Fermentation and Food Biomanufacturing, Jiangnan University, Wuxi, China; ^4^Department of Nutrition, Affiliated Hospital of Jiangnan University, Wuxi, China; ^5^School of Life Science and Health Engineering, Jiangnan University, Wuxi, China

**Keywords:** non–small-cell lung cancer, tumor microbiota, *Stenotrophomonas maltophilia*, cell signaling pathway, inflammation, high-throughput sequencing

## Abstract

**Introduction:**

Lung cancer is the leading cause of cancer death worldwide, and lung adenocarcinoma (LADC) is the most common lung cancer. Lung cancer has a distinct microbiome composition correlated with patients’ smoking status. However, the causal evidence of microbial impacts on LADC is largely unknown.

**Methods:**

We investigated microbial communities’ differences in Formalin-Fixed Paraffin-Embedded tissues of ever-smoke (*n* = 22) and never-smoke (*n* = 31) patients with LADC through bacterial 16S rRNA gene high-throughput sequencing. Then nitrosamines 4-(methylnitrosamino)-1-(3-pyridyl)-1-butanone (NNK)-induced lung cancer mouse model and A549 cells were used to study the effect of *Stenotrophomonas maltophilia* (*S. maltophilia*) in LADC.

**Results and Discussion:**

We found a significant increase of genus *Stenotrophomonas* in LADC tissues of patients with primary tumor size greater than 3 cm and never-smoker patients. We further found that intratracheal infection with *S. maltophilia* promoted tumor progression in the NNK-induced lung cancer mouse model. We performed RNA-seq analysis on lung tissues and found that *S. maltophilia* treatment drove inflammation and upregulated tumor associated cell signaling, including Apelin signaling pathway. Mechanistically, histone deacetylase 5 (HDAC5) gene expression was significantly upregulated in *S. maltophilia* treated groups, and was required for *S. maltophilia* induced cell proliferation and migration in LADC cell line A549. Therefore, we provide in vivo and in vitro evidence to demonstrate that *S. maltophilia* promotes LADC progression, in part, through HDAC5.

## Introduction

Lung adenocarcinoma (LADC) accounts for about 40% of all lung cancer, which is the predominant cause of cancer death worldwide (Sung et al., 2020). Adenocarcinoma of the lung belongs to non-small cell lung cancer (NSCLC), which usually occurs in the lung periphery and evolves from the mucosal glands. Although smoking tobacco is the leading driver of any lung cancer, including LADC (Schuller, 2002; Kenfield et al., 2008), only 15% of smokers will develop lung cancer (Samet et al., 2009), and the non-smoking-related etiology and carcinogenesis remain poorly understood (Jemal et al., 2018). As is well-known, primary tumor size is a significant prognostic factor in lung adenocarcinoma patients. Tumor size arises as a more important prognostic factor, from ≤1 cm to 7 cm, each centimeter separates tumors with a significantly different prognosis, and the 3-cutoff point separates T1 from T2 tumors (Detterbeck et al., 2016; Rami-Porta et al., 2017; Kim et al., 2019).

Mounting evidence suggests lung cancer is associated with many infectious diseases, such as COPD, tuberculosis, HIV, and *Chlamydia* infections (Chaturvedi et al., 2010; Yu et al., 2011; Dima et al., 2019; Cribbs et al., 2020). Meanwhile, lung infections destroy bronchial epithelial cells, creating a vicious circle (Huang and Shi, 2019). Studies have proved that the bacterial composition of bronchoalveolar fluid in lung cancer patients is different from that in benign disease patients. The microbiome composition of lung cancer in the lower airway sample is more similar to that of the buccal sample, which enriched with *Veillonella*, *Streptococcus*, *Prevotella*, and *Rothia* is related to lung cancer stage and prognosis (Lee et al., 2016; Tsay et al., 2020). Inhalation of antibiotics can reduce the lung colonization of melanoma (Le Noci et al., 2018).

In recent years, studies have proved that every tumor type has a distinct microbiome composition, and these intratumor bacteria are mostly intracellular and are present in both cancer and immune cells, suggesting an association with tumor development and clinical features (Nejman et al., 2020). The intratumor microbiota is a crucial mediator in tumor progression and migration. Studies have found that the tumor-resident microbiota can promote lung metastatic colonization in breast cancer through the upregulation of fluid shear stress pathway (Fu et al., 2022). Administration of bacteria through tail vein impairs tumor chemosensitivity (Yu et al., 2017) and promotes tumor progression (Le Noci et al., 2018; Parhi et al., 2020). However, the composition of the human LADC microbiome and the role of distinct altered bacterial species in lung cancer are still unknown.

In this study, we analyzed the differences in microbial compositions in ever-and never-smokers with LADC. Then we focused on the *Stenotrophomonas* genus, whose abundance correlated with the smoking status and primary tumor size. *Stenotrophomonas maltophilia* is the only species that infect humans, so we investigated the role of its type strain *S*. *maltophilia* (ATCC#13637) on the progression of lung cancer induced by nitrosamines 4-(methylnitrosamino)-1-(3-pyridyl)-1-butanone (NNK) in carcinogen-sensitive mouse strains A/Jmice. Further, we explored the potential mechanism of action of *S*. *maltophilia*. These findings suggest that a higher abundance of *S*. *maltophilia* is a risk factor in LADC and provide novel insights to evaluate cellular and molecular targets from the perspective of intratumor bacteria.

## Results

### Clinical characterization of lung adenocarcinoma patients

A total of 53 LADC samples were included in this study. These patients were admitted to the hospital and underwent surgery between January 2014 and December 2015. None of the patients in this study received antibiotics or chemotherapy before surgery. We collected information on gender, age, Primary tumor dimension, Lymphatic metastasis, clinical stage and followed the patient’s survival as of January 17, 2021. The mean age of the total cohort was 62.2 years, with 50.6% men and 41.5% smokers. The proportion of men in the ever-smokers group was higher than in the never-smokers group (94.1 vs. 31.9) because women rarely smoke. For primary tumor size, 35 patient tumor size is less than 3 cm, 18 patient tumor size is higher than 3 cm. In total, 16 samples belonged to the I–II stages and 6 samples to the III–IV stages in ever-smokers, while 24 samples belonged to the I–II stages and 7 samples to the III–IV stages in never-smokers (Table 1). According to the survival curve of the patients, we found that smoke might be a negative prognostic factor in LADC patients, but this was not statistically significant (Supplementary Figure S1A).

**Table 1:** Clinical characteristics of the lung adenocarcinoma patients.

Characteristic	Ever-smokers (*n* = 22)	Never-smokers (*n* = 31)	*P* value
Age, mean ± SD	66.77 (±6.597)	62.64 (±9.138)	ns
Overall survival, mean ± SD	62.86 (±27.692)	67.77 (±18.599)	ns
Sex, male	19	9	*p* < 0.001
Stage, n (%)			
I	14 (64%)	18 (58%)	ns
II	4 (18%)	6 (19%)	ns
III	4 (18%)	7 (23%)	ns
IV	0	0	

### Different clinical feature lung adenocarcinoma patients have distinct lung tumor microbial compositions

**Figure 1:**
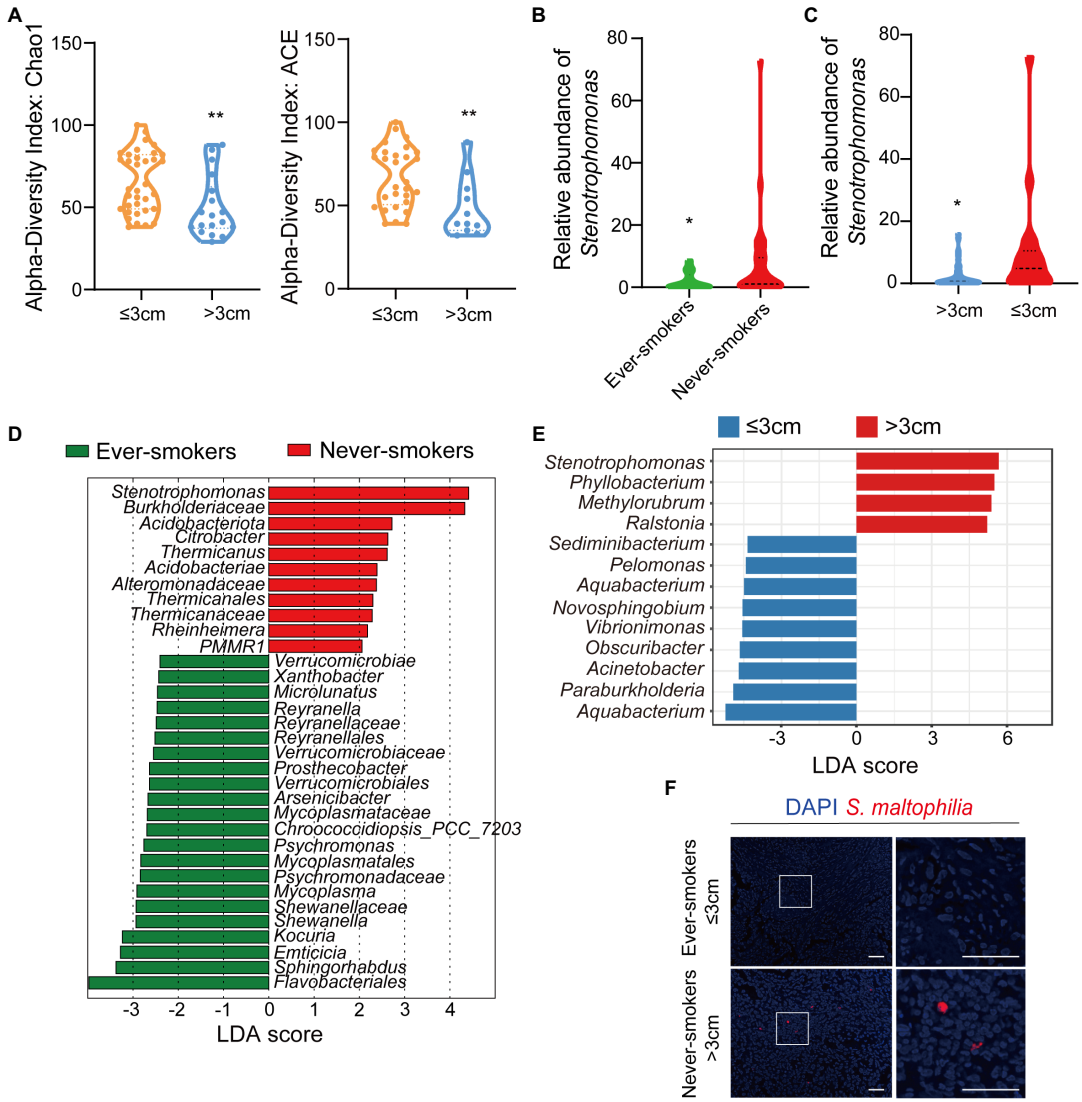
The microbiota was changed in lung tissues between the ever-smoker and never-smoker lung adenocarcinoma patients. **(A)** Taxonomic alpha-diversity calculated with the Chao index (*p* = 0.0057) and Ace index (*p* = 0.0058) between primary tumor size ≤3 cm and > 3 cm groups. **(B)** Relative abundances of *Stenotrophomonas* in the microbiota of ever-smoke and never-smoke groups. **(C)** Relative abundances of *Stenotrophomonas* in the microbiota of ≤3 cm and > 3 cm groups. **(D)** Linear discriminant analysis. Differential abundance of taxa was ranked according to their effect size between the Ever-smokers and Never-smoker groups. **(E)** Linear discriminant analysis. Differential abundance of taxa was ranked according to their effect size between primary tumor size ≤3 cm and > 3 cm group. The selection of discriminative taxa between groups was based on an LDA score cutoff of 3.0 and differences in the relative abundances of taxa (converted to log base 10) were statistically determined based on a Kruskal-Wallis and pairwise Wilcoxon tests. A value of *p* < 0.05 and a score ≥ 2.0 were considered significant. The length of the histogram represents the LDA score; i.e., the degree of influence of species with significant differences between different groups. **(F)** Representative FISH images of LADC tissue sections using a fluorescent probe specific to *Stenotrophomonas*. *p < 0.05, **p < 0.01 by Student’s t-test.

To analyze the composition of the tissue microbial profile, we successfully sequenced the 16S rRNA gene in Formalin-Fixed Paraffin-Embedded (FFPE) tissue samples from ever-smokers (*n* = 22) or never-smokers (*n* = 31) LADC. Firstly, we found alpha diversity metrics including Shannon, Chao1, ACE, and Simpson indexes according to amplicon sequence variants (ASVs) were comparable between two groups (Supplementary Figure S1B). We also visualized the Bray-Curtis distance of beta diversity by principal coordinate analysis (PCoA) between ever-smokers and never-smokers (*p* > 0.05, Supplementary Figure S1C). But we found that Chao1 and ACE index between primary tumor size ≤3 cm and > 3 cm group is significant (Figure 1A).

Then, we conducted the taxonomic assignment to build the microbial abundance profile. The two groups were both enriched by Proteobacteria, as the dominant phylum, followed by Actinobacteriota, Firmicutes and Bacteroidetes (Supplementary Figure S2), which was in consistent with the previous report (Nejman et al., 2020). We observed a significant increase of Acidobacteriota (*p* = 0.046912) in LADC tissues of never-smokers than ever-smokers. We identified in total 60 bacterial classes, 25 orders, 30 families, and 35 genera with a relative abundance higher than 0.1%. We also performed the analysis at class, order, family, and genera levels (Supplementary Figures S3, S4). At the genus level, the tumor microbiota of LADC in the ever-smokers was enriched with *Escherichia_Shigella* and *Sphingomonas*, but did not reach statistical significance (Supplementary Figure S5). While *Stenotrophomonas*, *Ralstonia*, and *Corynebacterium* were identified to be enriched in the never-smokers. Among them, *Stenotrophomonas* was more than 3.9 fold higher in lung tumors of never-smokers (*p* < 0.05, Figure 1B). We also found a significant increase of *Stenotrophomonas* in LADC tissues of primary tumor size >3 cm than ≤3 cm groups (Figure 1C).

Next, we used the LEfSe algorithm to identify the critical bacterial taxa that account for the differences between smoking status or primary tumor size. LEfSe estimates the effect size of significantly different abundant of taxa and ranks them represented by the length of each bar (Segata et al., 2011). We found family Verrucomicrobiaceae and its higher taxonomies, including class Verrucomicrobiae and order Verrucomicrobiales, were significantly enriched in ever-smokers (Figure 1D). There was an increase in the family Burkholderiaceae in never-smokers compared with ever-smokers. Moreover, LADC microbiota of never-smokers was enriched in Acidobacteriota and *Stenotrophomonas*, in line with phylum and genus level observations (Figure 1D). We also found that the genus *Stenotrophomonas* was enriched in the group of primary tumor size higher than 3 cm patients through LEfSe analysis (Figure 1E). There was a linear correlation between the relative abundance of *Stenotrophomonas* and primary tumor size (Supplementary Figure S6). To validate the presence of *Stenotrophomonas* in LADC tissues, we used FISH with a specific probe against its 16S rRNA. We found the presence of *Stenotrophomonas* DNA within LADC tissues in never-smokers, but not in that of ever-smokers (Figure 1F). These results indicates that *Stenotrophomonas* could be identified as biomarker in LADC.

### Intratracheal inoculation of *Stenotrophomonas maltophilia* promotes lung cancer progression

Although various studies have revealed that microbiome contributes to tumor induction and progression (Dapito et al., 2012; Li et al., 2016, 2019; Le Noci et al., 2018; Jin et al., 2019; Tsay et al., 2020; Fu et al., 2022), the causal relationship between *Stenotrophomonas* and lung cancer remains largely unknown. *S*. *maltophilia*, the only species of *Stenotrophomonas* that infects humans, is considered a “newly emerging pathogen of concern” (An and Berg, 2018). To investigate whether *S*. *maltophilia* could colonize in the lung, we harvested lung lobes and cultured the lung homogenate in the nutrient broth after 48 h of bronchial injection of *S*. *maltophilia* in mice. We visualized and validated the colonizing ability of *S*. *maltophilia* in lung lobes by 16S rRNA gene sequencing (Supplementary Figure S7).

**Figure 2:**
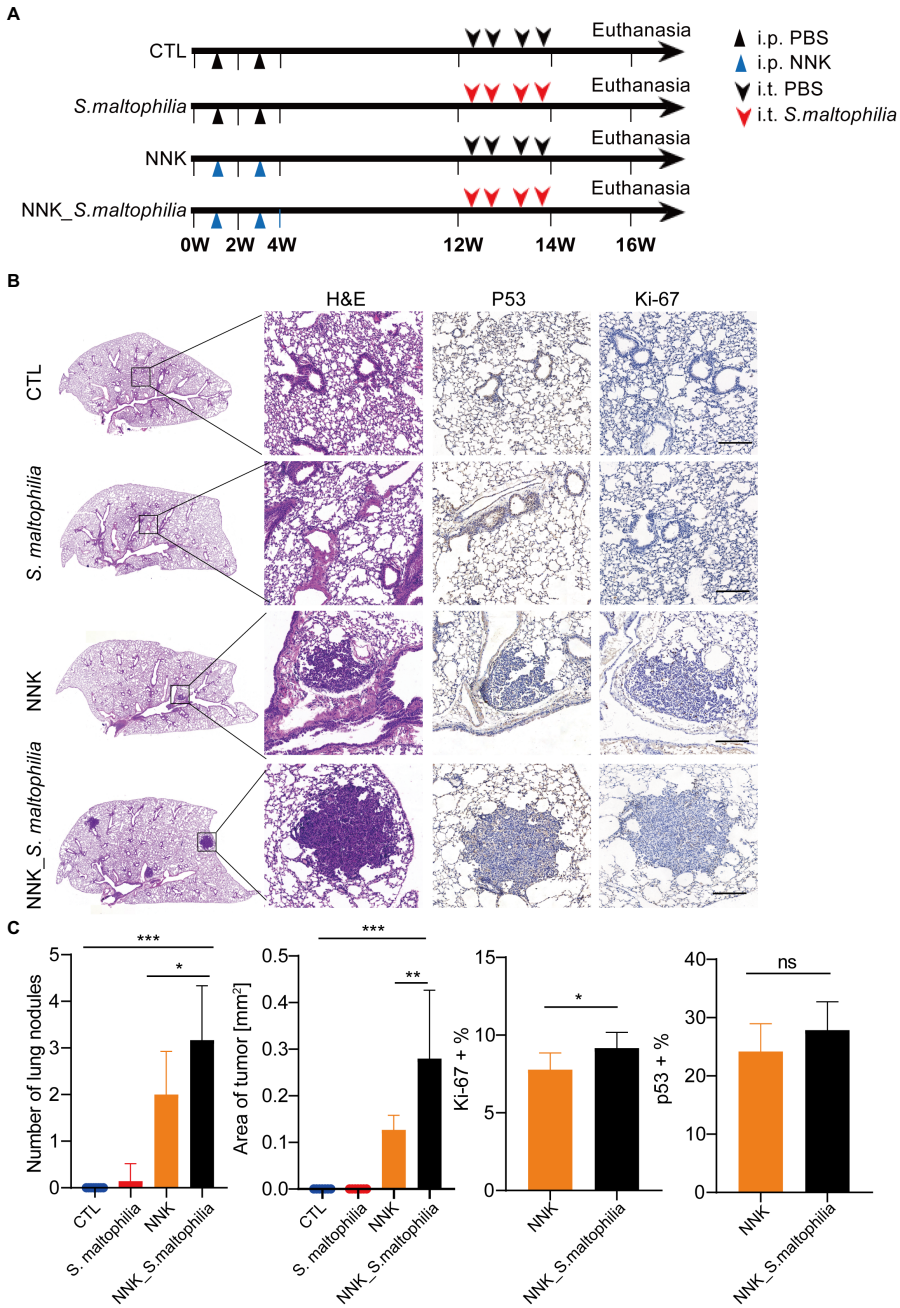
*Stenotrophomonas maltophilia* promotes lung cancer progression in mice. **(A)** Study design and diagram. **(B)** H&E staining, IHC staining of p53 and Ki-67 in lung tissues. Scale bar = 100 μM. **(C)** The quantification of nodules on the lung surface, primary tumor area (mm^2^), and percentage of p53 and Ki-67 positive area. Results are expressed as the mean ± SEM. *p < 0.05, **p < 0.01, ***p < 0.001 by Student’s *t*-test. For each experiment, *n* = 5–8 mice/group. ns, no significance.

To explore whether *S*. *maltophilia* contributes to lung cancer progression, we compared tumor development between mice intratracheal inoculated with *S*. *maltophilia* and their control A/J mice in lung cancer model induced by NNK (Akopyan and Bonavida, 2006; Figure 2A). We found that NNK treatment affected the weight gain in mice, but not *S*. *maltophilia* treatment (Supplementary Figure S8). Compared with the NNK model group, NNK_ *S*. *maltophilia* group displayed heavy tumor burden, with the increased number of lung nodules and area of tumor (Figures 2B,C). At the cellular level, although NNK_ *S*. *maltophilia* group did not affect P53 expression, it exhibited increased tumor cell proliferation as demonstrated by immunohistochemical (IHC) analysis of Ki-67 staining (Figure 2C). Together, these results indicate that *S*. *maltophilia* plays a profound role in promoting tumor development in NNK induced lung cancer model.

### Intratracheal inoculation of *Stenotrophomonas maltophilia* leads to a distinct transcriptional profile in lung tissues

**Figure 3:**
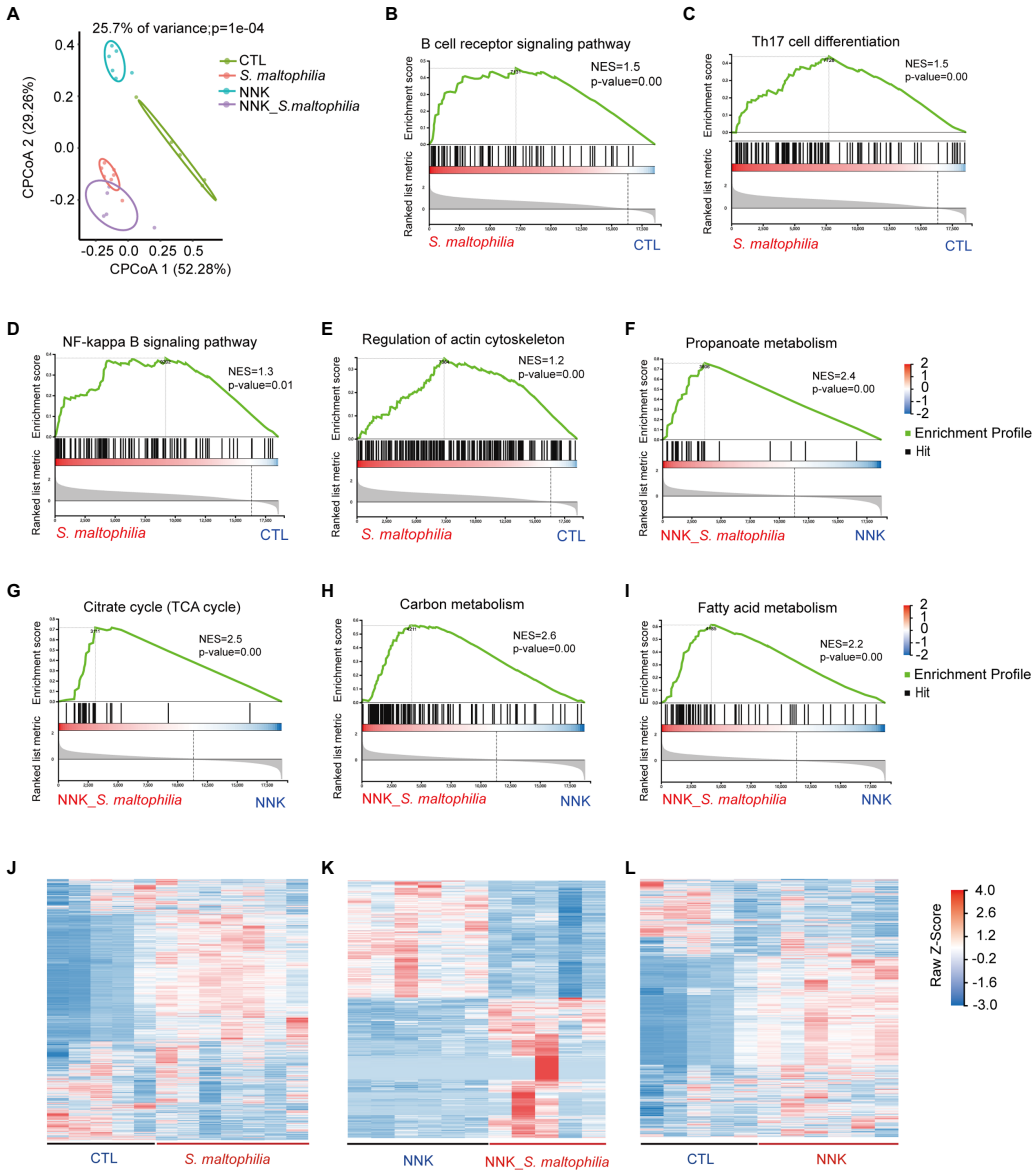
*Stenotrophomonas maltophilia* modulates gene expression in lung tissues by RNA-seq analysis. **(A)** Constrained PCoA clustering analysis of RNA-seq data. **(B–I)** GSEA Plot for the B cell receptor signaling pathway, Th17 cell differentiation, NF-kappa B signaling pathway, regulation of actin cytoskeleton, Propanoate metabolism, Citrate cycle (TCA cycle), Carbon metabolism, and Fatty acid metabolism, respectively. **(J)** Heatmap of different genes in lung tissues of *S*. *maltophilia* group compared with CTL group. **(K)** Heatmap of different genes in lung tissues of NNK_*S*. *maltophilia* group compared with NNK group. **(L)** Heatmap of different genes in lung tissues of NNK group compared with CTL group.

To investigate the underlying mechanisms of *S*. *maltophilia* on lung cancer progression, we characterized the transcriptional profile of lung tissue using RNA sequencing (RNA-Seq). Constrained Principal Co-ordinates Analysis (CPCoA) clustering analysis showed that the CTL and NNK groups were clearly separate, while two *S*. *maltophilia* treatment groups were relatively close to each other (Figure 3A). These results indicate that the NNK and *S*. *maltophilia* treatment groups had distant gene expression signatures.

Gene set enrichment analysis (GSEA) revealed 34 overrepresented gene sets in *S*. *maltophilia* group compared to CTL group based on normalized gene set enrichment scores (NES; Supplementary Figure S9). Among them, various inflammatory pathways, including B cell receptor signaling pathway, Th17 cell differentiation, NF-kappa B signaling pathway, as well as regulation of actin cytoskeleton were significantly enriched in *S*. *maltophilia* group (Figures 3B–F). Sixteen gene sets were enriched in NNK_ *S*. *maltophilia* group compared with NNK group (Supplementary Figure S10). Interestingly, metabolism associated with tumor reprogramming, including propanoate metabolism, citrate cycle (TCA cycle), carbon metabolism, and fatty acid metabolism, were significantly enriched in NNK_ *S*. *maltophilia* group (Figures 3G–I). These data suggest that *S*. *maltophilia* may trigger inflammatory response, regulate cytoskeleton, and promote cancer metabolism.

Next, differentially expressed genes (DEGs) were identified through pairwise comparisons of groups. Compared with the CTL group, the number of DEGs in the *S*. *maltophilia*, NNK, and NNK_*S*. *maltophilia* groups was 3,786 (1832/1954; upregulated and downregulated DEGs, respectively), 3,373 (1,335/2038), and 2,283 (1,163/1120) respectively (Figures 3J–L; Supplementary Figure S11; Supplementary Table S1). A total of 1,034 DEGs were identified between NNK and NNK_S. *maltophilia* groups, among them 564 genes were upregulated in the lung tissues of NNK_*S*. *maltophilia* group and 470 genes were downregulated (Figure 3K). NNK_S. *maltophilia* group and *S*. *maltophilia* groups displayed similar gene expression profiles, and only 286 DEGs (the lowest number among all comparisons) were identified between these two groups (Supplementary Table S1). Additionally, we were able to identify 322 common DEGs between the CTL-*vs*-*S*. *maltophilia*, and NNK-*vs*-NNK_*S*. *maltophilia* (Supplementary Figure S12; Supplementary Table S2).

**Figure 4:**
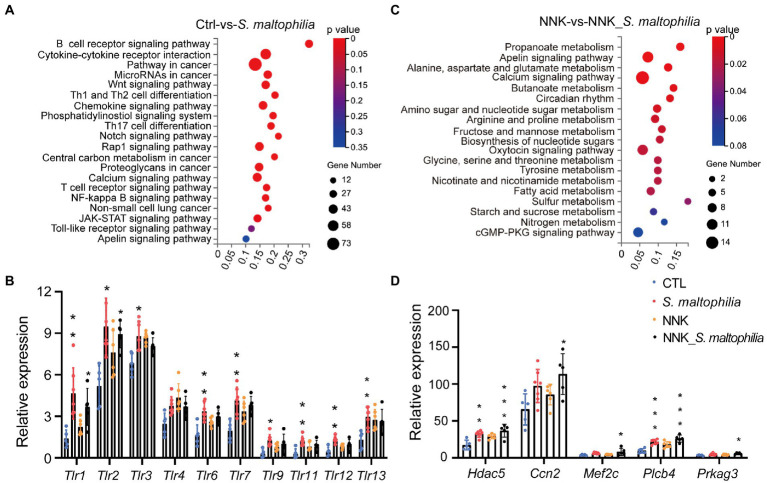
*Stenotrophomonas maltophilia* modulates gene expression in lung tissues by RNA-seq analysis. **(A)** KEGG pathway enrichment analysis of upregulated genes of RNA-seq data between CTL and *S*. *maltophilia* groups. **(B)** Relative expression of Toll-like receptors. **(C)** KEGG pathway enrichment analysis of upregulated genes of RNA-seq data between NNK and NNK_S. maltophilia groups. **(D)** Relative expression of Apelin signaling pathway genes. *p < 0.05, **p < 0.01, ***p < 0.001, compared with CTL.

We next calculated the specific KEGG pathway enrichment of upregulated DEGs in *S*. *maltophilia* treated groups. Non-small cell lung cancer, Pathways in cancer and Ras signaling pathway enrichment confirmed that NNK induced lung cancer in A/J mice (Supplementary Figure S13). Although *S*. *maltophilia* treatment did not trigger lung cancer development in A/J mice, we found that pathway in cancer, MicroRNAs in cancer, Central carbon metabolism in cancer, and Non-small cell lung cancer was enriched in *S*. *maltophilia* group compared with CTL group (Figure 4A). KEGG pathway analysis also revealed that mice treated by *S*. *maltophilia* turned on many immunity-related signals, including the B cell receptor signaling pathway, Th1 and Th2 cell differentiation, Chemokine signaling pathway, Th17 cell differentiation, T cell receptor signaling pathway, NF-kappa B signaling pathway, and Toll-like Receptor signaling pathway (Figure 4A). Several Toll-like Receptor (TLR) genes were highly upregulated in *S*. *maltophilia* groups compared to CTL or NNK group (Figure 4B). Consistent with GSEA, we found propanoate metabolism and fatty acid metabolism was enriched in NNK_ *S*. *maltophilia* group compared with NNK group (Figure 4C).

The Apelin signaling pathway was enriched in the *S*. *maltophilia* group compared with the CTL group and in NNK_ *S*. *maltophilia* group compared with NNK group (Figures 4A,C). We found that 11 genes that belonged to the Apelin signaling pathway were upregulated in NNK_*S*. *maltophilia* group (Supplementary Table S3). Interestingly, five of them (*Hdac5*, *Ccn2*, *Mef2c*, *Plcb4*, *Prkag3*) were overexpressed in both *S*. *maltophilia* and NNK_*S*. *maltophilia* group (Figure 4D). Among them, HDAC5 (histone deacetylase 5), as a member of the class IIa family of HDACs, is a well-known oncogene in numerous cancer types, including lung cancer (Zhong et al., 2018; Yang et al., 2021). These results suggest that *S*. *maltophilia* may promote lung cancer progression by driving inflammation, regulating tumor cell signaling and metabolism at the transcriptional level.

### *Stenotrophomonas maltophilia* promoted cell proliferation and cell migration of lung epithelial cells

**Figure 5:**
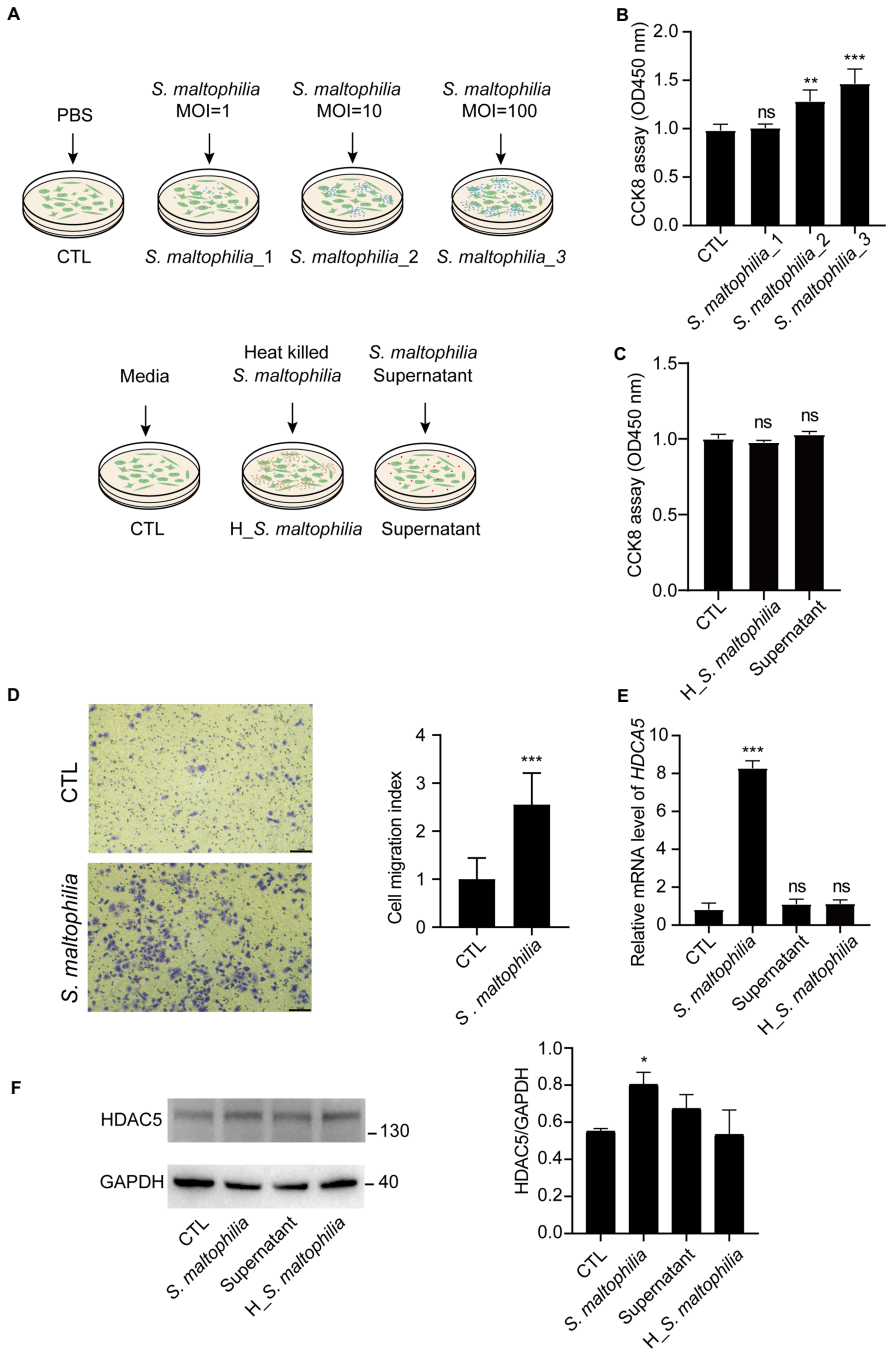
*Stenotrophomonas maltophilia* promoted cell and migration in lung epithelial cells. **(A)** Schematic experimental design for two *in vitro* experiments. For all conditions, A549 cells were exposed for 4 h and then harvested for RNA isolation. Experiment 1: Cells were infected with *S*. *maltophilia* (ATCC #13637) at three different multiplicity of infection (MOI) of 1, 10, and 100 for 4 h. Experiment 2: exposure to media alone, *S*. *maltophilia* (heat-killed), and supernatant. **(B)** Effect of *S*. *maltophilia* on A549 cell viability. **(C)** Effect of heat-killed *S*. *maltophilia* and supernatant on A549 cell viability, ns: no significance. **(D)** Effects of *S*. *maltophilia* on A549 cell line migration. **(E)** Effects of *S*. *maltophilia* on HDAC5 gene expression in A549 cells. **p < 0.01 compared with CTL, ***p < 0.001 compared with CTL. **(F)** Western blot and statistical analysis of HDAC5 expression in A549 cells. *p < 0.05. ns, no significance.

To explore the possible mechanism of *S*. *maltophilia* in promoting LADC in mice, we performed *in vitro* experiments using cultured A549 cells exposed to the microbial products (Figure 5A). At first, we used three concentrations of bacteria for the *in vitro* experiment: multiplicity of infection (MOI) of 1, 10, and 100. We found that compared with CTL, 4 h pre-incubation with *S*. *maltophilia*_1 and *S*. *maltophilia*_2 significantly promoted A549 cell proliferation (Figure 5B). Then to explore the potential factors of *S*. *maltophilia* that contribute to tumor cell proliferation, we exposed A549 cells to heat killed or the supernatants from viable bacteria. The result showed that both of them did not promote cell proliferation (Figure 5C). These data suggest that viable *S*. *maltophilia* may need to communicate with the cell to stimulate its proliferation.

**Figure 6:**
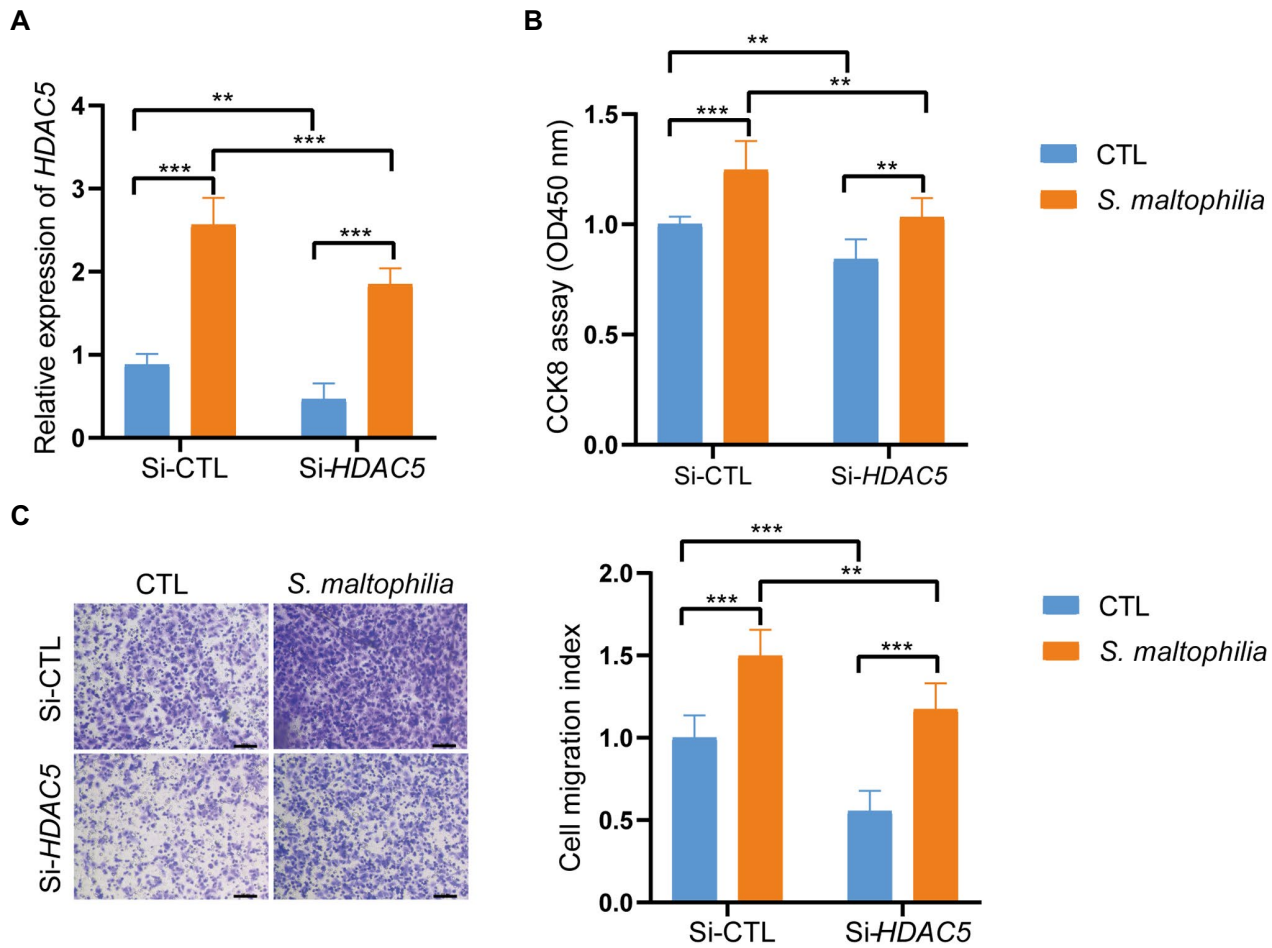
Inhibition of HDAC5 suppressed lung cancer cellular functions. Lung cancer cells A549 were transfected with si-HDAC5 or negative control (si-NC). **(A)** The HDAC5 expression levels were detected by qRT-PCR. **(B)** CCK-8 assay was used to test the cell proliferation. **(C)** Transwell migration assay was used to test the cell migration ability. *p < 0.05, ***p < 0.001 by Student’s t-test.

*Stenotrophomonas maltophilia* treatment also promoted cell migration in A549 cell lines (Figure 5D). To verify the transcriptome results in mouse lungs, we performed qPCR assays. We found that pre-exposure to *S*. *maltophilia* significantly increased the mRNA expression of HDAC5 in A549 cells (Figure 5E). We further confirmed the up-regulated expression of HDAC5 in A549 cells treated with *S*. *maltophilia* by western blot (Figure 5F). Considering the tumor-promoting role of HDAC5, we further reasoned if HDAC5 silencing would abrogate the *S*. *maltophilia* induced cell proliferation and migration in lung cancer cells. We verified that HDAC5-specific siRNA markedly downregulated its gene expression, as well as inhibited cell proliferation and migration in A549 cells (Figures 6A–C). Further, we found that the cell proliferation and migration stimulated by *S*. *maltophilia* were also attenuated by HDAC5 interference (Figures 6A–C). These findings demonstrate that *S*. *maltophilia* promotes lung cancer cell proliferation and migration partially through HDAC5.

## Discussion

Recently, there has been increasing awareness that human tumors contain a significant amount of viable commensal microbiota (Banerjee et al., 2018; Riquelme et al., 2019; Nejman et al., 2020; Dumont-Leblond et al., 2021). Whether these microbes are passengers or drivers of tumor progression is an intriguing question that emerges. But a detailed and comprehensive analysis of the microbial ecosystem of the pathologic and lung cancer tissues remains incompletely studied. In this study, firstly, we represent for the report to characterize the microbiota composition of 53 samples from human LADC tissue. We performed 16S rRNA sequencing of the LADC and took measures to control for contamination. We explored the distinct microbiome composition inside the LADC, and uncovered significant associations between the bacteria and transcriptional changes in lung cancer cells that may be relevant for lung cancer pathogenesis. Overall, we observed significant intratumor microbiome dysbiosis in LADC patients with a different clinical feature. We found although there was no significant difference in tumor microbial alpha and beta diversity between ever-smokers and never-smokers LADC, representatives of the phyla Acidobacteriota and genus *Stenotrophomonas* were predominant in never-smokers. And we also found genus *Stenotrophomonas* was significantly enriched in the group of patients with primary tumor size higher than 3 cm patients. However, due to the limited number of cases, there is still no systematic evaluation of risk factors or clinic features associated to a different microbiome microenvironment.

Most recently, a higher abundance of *S*. *maltophilia* was found in hepatocellular carcinoma microbiota of the patients with cirrhosis, which induced the senescence of hepatic stellate cells and promoted the process of hepatocarcinogenesis (Liu et al., 2022). Notably, we demonstrated for the first time that the upregulation of HDAC5 caused by *S*.*maltophilia* infection is associated with tumor progression, supporting a causal role in the process of lung tumor growth.

Study have suggested that chronic colonization *C*. *difficile* is a potential driver of colorectal cancer in patients (Drewes et al., 2022). Coincidentally, *S*. *maltophilia* is an opportunistic pathogen that is multidrug resistant and causes a variety of human infections and forms biofilms in infected patients (Brooke, 2021), the most common *S*. *maltophilia*-associated human infections are bacteremia and respiratory infections (Batra et al., 2017). *S*. *maltophilia* is intrinsically resistant to a wide range of antibiotics, including β-lactams, carbapenems, fluoroquinolones, tetracyclines, chloramphenicol, aminoglycosides, polymyxins, macrolides, and TMP-SMX (Brooke, 2012), Once infected, it will colonize for a long time and is challenging to eliminate with antibiotics. In a previous study, variation in replication and persistence of *S*. *maltophilia* can be seen with clinical strains in A/J mice (Rouf et al., 2011), which demonstrated that our model is suitable for studying the function of *S*. *maltophilia*. In our study, we found that lung cancer mice treated with *S*. *maltophilia* have increased tumor burden compared with those treated with PBS. Future work identifying the effect of *S*. *maltophilia* colonization, duration, and toxin production on cancer cell interactions in humans will be needed to determine the tumorigenic risk to patients.

Histone deacetylase 5 is a class II HDAC (de Ruijter et al., 2003), which is expressed in lung, brain, myocardium, skeletal muscle, and placenta, and accumulating evidence indicates that it has variable expression and functions in different types of tumors (Stypula-Cyrus et al., 2013; Zhong et al., 2018; Oltra et al., 2020). The functions of HDAC5 in tumorigenesis have been investigated in a variety of cancers, and HDAC5 was also shown to promote cell proliferation, invasion and metastasis in cancer (Chen et al., 2014; Liu et al., 2014; Cao et al., 2016, 2017; Zhong et al., 2018). PCR and immunohistochemical analyses have shown that HDAC5 was highly expressed in the cytoplasm of malignant epithelial cells, and HDAC5 expression was positively associated with distant metastasis and lymph node metastasis (Li et al., 2016). HDAC5 was also shown to promote cell invasion and metastasis in lung cancer (Gong et al., 2020). Our data point to mechanisms of *S*. *maltophilia* induced tumorigenesis, including increased Apelin signaling pathway, which HDAC5 gene is the most important. Consistent with our data from the mouse model, we also found *S*. *maltophilia* induced HDAC5 expression in lung cancer cells. These bacteria may affect the host by shedding different microbial bioactive molecules, because heat-killed *S*. *maltophilia* and supernatants did not cause proliferation and migration and did not upregulate the HDAC5 gene expression of cancer cells *in vitro*.

We acknowledge several limitations. Our sample size is not large enough, and we only detected the bacterial species with the widely used V4 amplification from formalin-fixed paraffin embedded lung tumors. The coverage and resolution of the detection of *S*. *maltophilia* species may not be fully achieved. Our experiment did not uncover which specific components of the bacteria are responsible for tumor progression. We only verified the functions of HDAC5 in *S*. *maltophilia* stimulated cell proliferation and migration. There might be other mechanisms responsible for the tumor promoting effect of *S*. *maltophilia*.

In conclusion, we profiled the differences of the composition of microbiota in LADC using 16S rRNA gene high-throughput sequencing and found *Stenotrophomonas* increased in LADC tissues of patient with primary tumor size greater than 3 cm and never-smoker patients. Further *in vivo* and *in vitro* evidence demonstrate that *S*. *maltophilia* promotes cell proliferation and migration, as well as LADC progression, in part, through HDAC5.

## Materials and methods

### Patients and sample collection

Formalin-Fixed Paraffin-Embedded (FFPE) tissue samples of 53 patients diagnosed with LADC in the period from Jan 2014 to Dec 2015 were included. All procedures conformed to the Code of Ethics of the World Medical Association (Declaration of Helsinki) and complied with the guidelines of the Institutional Review Board of the Affiliated Hospital of Jiangnan University (IRB: LS2021072).

### Isolation and identification of microbiota in lung adenocarcinoma

DNA was extracted from each FFPE sample using the TIANquick FFPE DNA kit (Tiangen, #DP330-02, Beijing, China) according to the manufacturer’s instruction. In addition, we introduced 3 negative controls which are empty tubes that were processed together with the samples for sequencing. All negative controls were processed according to the same protocols.

The DNA was amplified by polymerase chain reaction (PCR) with a bacterial 16S rRNA gene V4 region universe primer pair (341F: 5’-ACT CCT ACG GGA GGC AGC AG-3′ and 806R: 5′-GGA CTA CHV GGG TWT CTA AT-3′; Zhong et al., 2018). Barcoded libraries were generated and the products were sequenced on the HiSeq2500 platform (BGI, Wuhan, China) using the PE300 module. Clean tags were assigned to ASVs using DADA2 (Divisive Amplicon Denoising Algorithm) software in the Quantitative Insights Into Microbial Ecology (QIIME) 2 package, and tags with ≥100% similarity were clustered to the same ASV. Representative ASVs were annotated using Silva_species_assignment_v138 reference database. The ASVs without annotation or annotated to polluted species were removed. Picrust2 (v2.2.0) was used for function predictions.

Common and specific ASVs among groups were compared and displayed in R 3.6.2 with “venn” package (v3.1.1) and Bray-Curtis similarities were calculated in R with “vegan” package (v3.5.1). Alpha diversity was applied to analyze the complexity of species diversity of a sample using Chao 1, ACE, Shannon, and Simpson indices. All indices of our samples were calculated with Mother (v1.31.2). Observed species and Chao 1 were selected to identify community richness, whereas Shannon was used to identify the community diversity. Beta diversity was calculated on both weighted and unweighted UniFrac using QIIME (v1.80). Partial least squares discrimination analysis (PLS-DA) was built using the mixOmics package (v3.2.1) in R. The differential abundance at the phylum, class, order, family, genus, and species levels between groups were performed using LEfSe, with *p* ≤ 0.01 and LDA ≥ 2.

### Bacteria

*Stenotrophomonas maltophilia* (ATCC #13637 = CGMCC#1.1788 = BCRC#10737 = DSM#50170 = LMG#958 = NBRC#14161 = NCTC#10257) were purchased from the China General Microbiological Culture Collection Center. *S*. *maltophilia* were cultured in nutrient broth (Solarbio #n8300, China) at 30°C in an aerobic chamber for 24 to 48 h.

### Animals and treatments

Female A/J mice aged 5 weeks were purchased from GemPharmatech Co., Ltd (Nanjing, China). All mice were housed in a specific-pathogen-free (SPF) environment with access to a standard chow diet and drinking water *ad libitum*. After a week of acclimation, mice were randomly divided into control group (CTL), bacteria solution (*S*. *maltophilia*) group, NNK treatment group (NNK, 100 mg/kg, biweekly for 4 weeks), and NNK + bacteria solution group (NNK_ *S*. *maltophilia*; *n* = 8 per group). The mice were injected intraperitoneally (i.p) or intratracheal inoculation (i.t) at the indicated doses and timepoints (Figure 2A). The intratracheal inoculation of *S*. *maltophilia* (1.5 × 10^6^ cfu/mL) were performed as described (DuPage et al., 2009). All mice were treated in accordance to the guidelines of the European Community (Directive 2010/63/EU) and the procedures were approved by the Committee of Ethics in Jiangnan University (#JN. No 20211130a0320401[504]).

### Histology and immunohistochemistry

The left lobe of the lung from the sacrificed mice was harvested and fixed by 4% paraformaldehyde. After embedding, sections were prepared and stained with hematoxylin–eosin. IHC staining was performed on unstained sections after antigens retrieval with citrate buffer (10 mM Sodium Citrate, 0.05%Tween 20, pH6.0; Jin et al., 2019). The following antibodies were used, including Ki-67 (1: 200 Abcam #ab16667, Cambridge, United Kingdom) and P53 (1: 2000 proteintech #60283-2-Ig, Chicago, United States), secondary antibody (MXB Biotechnologies #KIT-9922, Fuzhou, China). Digitally scanned images of stained slides were created with the Panorama MIDI (3DHISTECH Ltd., Budapest, Hungary).

### Cell culture, treatment, and viability assay

The A549 cell lines were kindly provided by Stem Cell Bank (Chinese Academy of Sciences). Cell were maintained in RPMI1640 (Gibco #11875093, Waltham, MA, United States) containing 10% FBS (Gibco #10099141C, Waltham, MA, United States), 1% Penicillin–Streptomycin-Amphotericin B Solution (Beyotime #C0224-100 ml, Shanghai, China), cultured in an incubator at 37°C under 95% air and 5%CO_2_. Cells were grown and 1 × 10^6^ cells plated in each well (6-well plates with 2 ml of media) and exposed them to different microbial challenges under *in vitro* experiments. Cells were infected with *S*. *maltophilia* (ATCC #13637) at three different multiplicy of infection (MOI) of 1, 10, and 100 for 4 h. Then the cells were washed twice, and incubated with fresh culture medium for 12 h. For heat inactivation, the bacteria were incubated at 60°C for 15 min. Cell viability was detected by a cell counting Kit-8 (Teyebio #TY0312, Shanghai, China) according to the manufacturer’s instruction.

A549 cells were chosen to perform further experiments. A549 cells (2 × 10^5^) were seeded in a 6-well tissue culture plate with 2 mL antibiotic-free RPMI-1640 medium supplemented with 10% FBS. When cells reached 60–80% confluence, the cells were transfected with HDAC5 siRNA, and control vector (Guangzhou RiboBio Co., Ltd., Guangzhou, China) using LipoRNAi™ Transfection Reagent (Beyotime, #C0535, Shanghai, China) according to the manufacturer’s specification. Then, A549 cells were incubated with the compound at 37°C in a CO_2_ incubator for 6 h. Following, the transfection mixture was replaced with fresh medium to culture for 48 h. Finally, the A549 cells were assayed using the appropriate protocol.

### Transwell migration assay

For Boyden chamber assays, cell suspension (1 × 10^5^cells) was placed into the upper compartment of a 8 μM pore size Transwell chamber (Merck #PTEP24H48, Billerica, MA, United States) in 24-well plate. In each well, serum-free medium was used in the top chamber, while medium containing 10%FBS were used in the bottom chamber. After 24 h culture, cells migrated to the bottom side of the membrane were fixed and stained with the 0.1% crystal violet solution. The migrated cells was counted in five randomly chosen fields per filter from triplicate filters per sample at ×400 magnification. The cell migration index was calculated as the number of cells able to migrate normalized to controls (mean ± SEM).

### Fluorescence *in situ* hybridization

FISH was conducted with the paraffin-embedded lung tissue according to the protocol described previously (Geller et al., 2017). Briefly, after deparaffinization and rehydration, the slides were incubated with 0.2 M HCl for 12 min at 37°C, and then were incubated with Triton at 37°C for 17 min. Slides were washed twice with PBS and incubated with lysozyme for 15 min at 37°C, and then they were hybridized with the probe specific to ASV4. Before visualization, DAPI was added to the slides. The *S*. *maltophilia* probe used for FISH was: GTC GTC CAG TAT CCA CTG C, 5′ modification: Cy3 (Liu et al., 2022). Then the images were captured using Confocal Laser Scanning microscopy (Carl Zeiss Microscopy, United States).

### RNA isolation and qRT-PCR analysis

RNAs were extracted from lung tissues and cells using Trizol reagent (Invitrogen, Waltham, United States) and were reversely transcripted into cDNAs. MultiScribe Reverse Transcriptase (Abm #G492, Canada) was used for cDNA synthesis, and then qRT-PCR was performed by using SYBR™ Select Master Mix (Thermo Fisher Scientific, Waltham, United States) according to the manufacturer’s protocols. Gene expression was measured relative to the endogenous reference gene β-actin using the comparative ΔCT method.

Sequences of the specific primer sets are as follows: HDAC5 (NM_001015053.2), forward, 5′-GTG ACA CCG TGT GGA ATG AG-3′; reverse, 5′- AGT CCA CGA TGA GGA CCT TG; β-actin (NM_001101.5), forward, 5’-CTC TTC CAG CCT TCC TTC CT-3′; reverse, 5’-AGC ACT GTG TTG GCG TAC AG-3′.

### Immunoblotting

Cells were lysed in RIPA buffer (Yeasen, Shanghai, China) and followed by 12% SDS-PAGE separation. Separated proteins were transferred onto polyvinylidene difluoride (PVDF) membranes (Merck Millipore, Billerica, MA, United States). The membranes were blocked by 5% bovine albumin in tris-buffered saline plus 0.1% Tween 20 for 1 h at room temperature. The membranes were probed with HDAC5 Antibody (Abcam, #ab55403, Cambridge, United Kingdom) and then probed with anti-rabbit IgG Antibody (Cell Signaling, #7074P2, Danvers, MA, United States). After washing with TBS-T, the membranes were visualized with SuperSignal West Pico PLUS substrate (Thermo Fisher Scientific Inc., Waltham, MA, United States). GAPDH Antibody (Cell Signaling, #5174S, Danvers, MA, United States) was used as a loading control.

### Library construction, RNA-seq, and data analysis

The total RNA isolated from each lung tissue sample was applied to RNA-Seq library preparation, by following the protocol described before (Zhu et al., 2018). Briefly, the mRNA was purified by oligo dT-attached magnetic beads and fragmented into small pieces, followed by first-strand and second-strand cDNA synthesis. After PCR amplification, purification, heat denaturation and cyclization, the final cDNA library was sequenced on the BGISEQ-500 platform (BGI-Shenzhen, China) using 50-bp single reads.

The sequencing data was filtered with SOAPnuke (v1.5.2; Li et al., 2008) by (1) Removing reads containing sequencing adapter; (2) Removing reads whose low-quality base ratio (base quality less than or equal to 5) is more than 20%; (3) Removing reads whose unknown base (“N” base) ratio is more than 5%, afterwards clean reads were obtained and stored in FASTQ format. The clean reads were mapped to the reference genome using HISAT2 (v2.0.4; Kim et al., 2015). Bowtie2 (v2.2.5; Langmead and Salzberg, 2012) was applied to align the clean reads to the reference coding gene set, then expression level of gene was calculated by RSEM (v1.2.12; Li and Dewey, 2011). Essentially, differential expression analysis was performed using the DESeq2 (v1.4.5; Love et al., 2014). with |log2 (Fold change)| > 0.4 and Q value ≤0.05. The heatmap was drawn by pheatmap (v1.0.8) according to the gene expression level calculated by RSEM (v1.2.12). GO (Gene Ontology) and KEGG (Kyoto Encyclopedia of Genes and Genome) enrichment analysis of annotated different expressed gene was performed by Phyper based on Hypergeomertric test. The significant levels of terms and pathways were corrected by Q value with a rigorous threshold (Q value ≤0.05) by Bonferroni.

### Statistical analysis

Data are expressed as the mean ± SEM (standard error of the mean). All experiments were repeated at least three times. Student’s two-tailed *t*-test was used for comparing differences between two groups. One-way or two-way ANOVA followed by Tukey–Kramer’s multiple-comparison test was used for multiple comparisons. Significance was set at *p* < 0.05. GraphPad Prism software (version 8.0, San Diego, United States) was used for statistical analysis.

## Data availability statement

The datasets presented in this study can be found in online repositories. The names of the repository/repositories and accession number (s) can be found at: https://nmdc.cn/, NMDC40015167. https://ngdc.cncb.ac.cn/gsa, GSA: CRA008629.

## Ethics statement

The studies involving human participants were reviewed and approved by the Institutional Review Board of the Affiliated Hospital of Jiangnan University. The patients/participants provided their written informed consent to participate in this study. The animal study was reviewed and approved by the Committee of Ethics in Jiangnan University.

## Author contributions

JS, YN, YG, and QY conceived and designed the study. JS, YN, QG, RL, and HC performed the experiments. JS, YN, QG, and RL performed the data analysis and bioinformatics analysis. JS wrote the manuscript. HC commented on the study. YG revised the manuscript. YG and QY approved submission of the manuscript. All authors contributed to the article and approved the submitted version.

## Funding

This work was supported by the grant from the Innovation Team of Wuxi Health and Family Planning Commission (CXTD2021005), Project of Taihu Talent Plan, and Qing Lan Project in Jiangsu Province.

## Conflict of interest

The authors declare that the research was conducted in the absence of any commercial or financial relationships that could be construed as a potential conflict of interest.

## Publisher’s note

All claims expressed in this article are solely those of the authors and do not necessarily represent those of their affiliated organizations, or those of the publisher, the editors and the reviewers. Any product that may be evaluated in this article, or claim that may be made by its manufacturer, is not guaranteed or endorsed by the publisher.

## Supplementary material

The Supplementary material for this article can be found online at: https://www.frontiersin.org/articles/10.3389/fmicb.2023.1121863/full#supplementary-material
