# Supplementary material for: Stenotrophomonas maltophilia promotes lung adenocarcinoma progression by upregulating histone deacetylase 5
Source: Front Microbiol. 2023 Feb 1;14:1121863. doi: 10.3389/fmicb.2023.1121863 (PMC9929947; doi:10.3389/fmicb.2023.1121863)
Supplement: Supplementary file 1 [file Data_Sheet_1.ZIP › Supplements/Supplementary_Material.docx]

Supplementary Material

*Stenotrophomonas maltophilia* promotes lung adenocarcinoma progression by upregulating HDAC5

**Jiyu Shen1†, Yalan Ni1,2†, Qijie Guan3, Rui Li2, Hong Cao4, Yan Geng5*, Qingjun You1,2***

*** Correspondence:** Qingjun You: [youqingjun@jiangnan.edu.cn](mailto:youqingjun@jiangnan.edu.cn)； Yan Geng, [gengyan@jiangnan.edu.cn](mailto:gengyan@jiangnan.edu.cn).

# Supplementary Figures


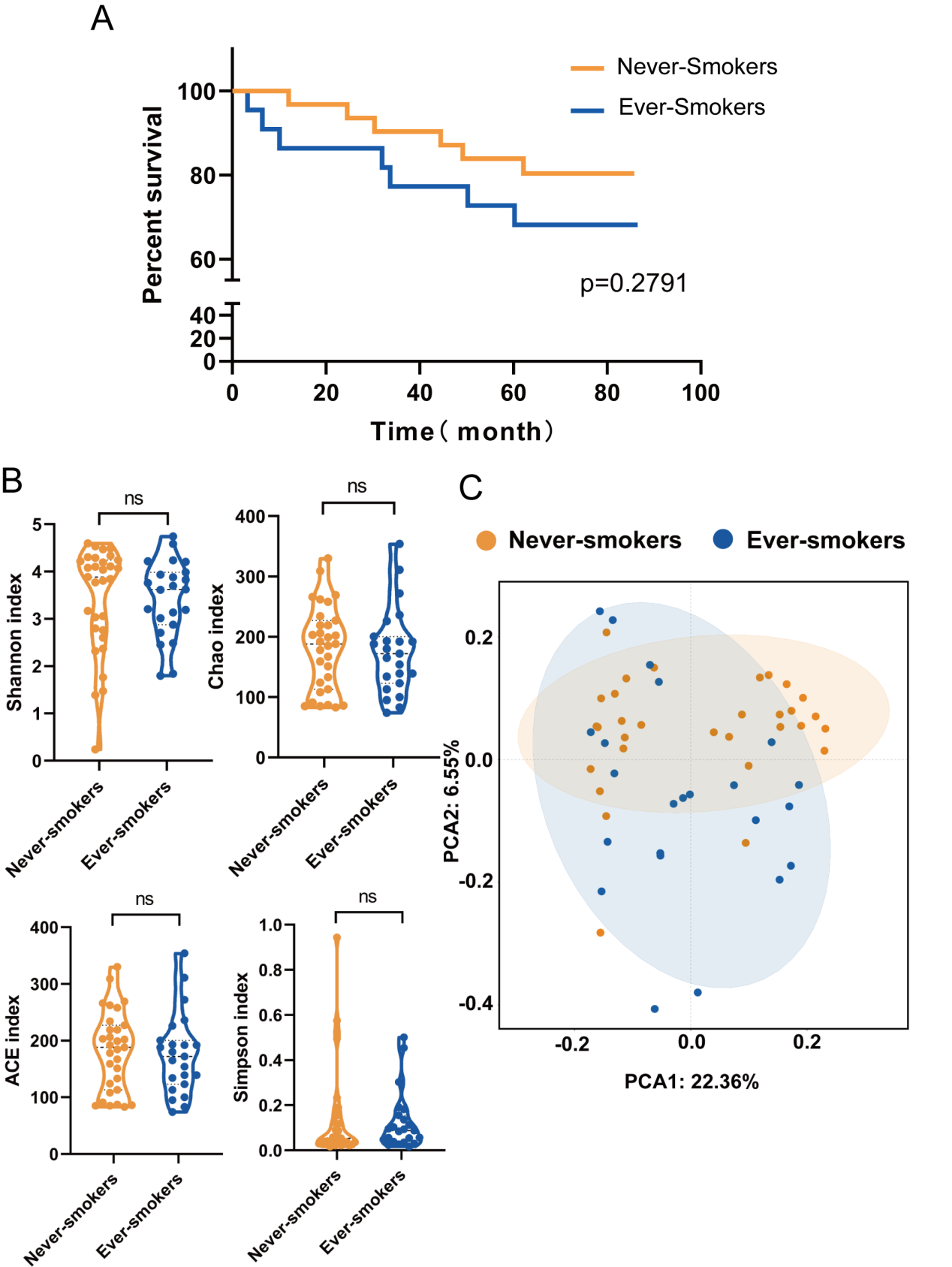


**Supplementary Figure 1.** The overall survival curves and lung microbial profiles of ever-smokers and never-smokers with lung adenocarcinoma. **(A)** Overall survival curves of patients according to the smoke status. **(B)** Taxonomic alpha-diversity calculated with the Shannon index, Chao index, Simpson index, Ace index, and Sobs index between ever-smokers and never-smokers groups (ns-nonsignificant). **(C)** PCoA plot based on the Bray-Curtis distance of the lung adenocarcinoma microbiome between ever-smokers and never-smokers group.


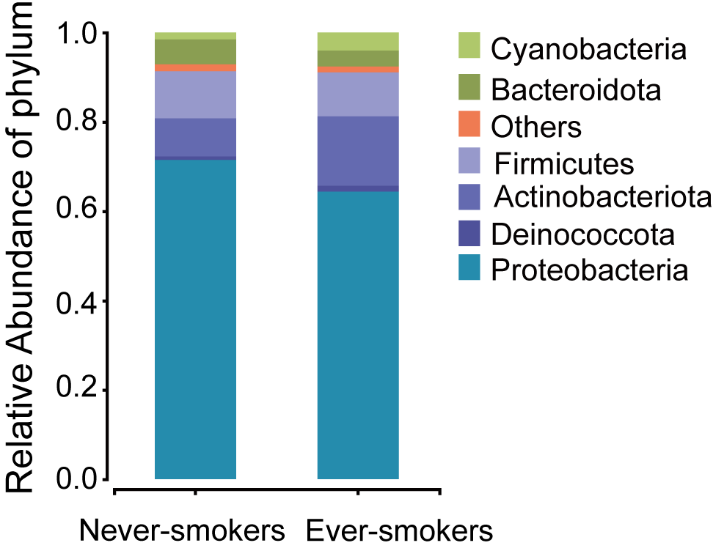


**Supplementary Figure 2.** Relative abundances of the dominant bacterial phylum in the microbiota of lung tissues in ever-smoke and never-smoke patients with lung adenocarcinoma.


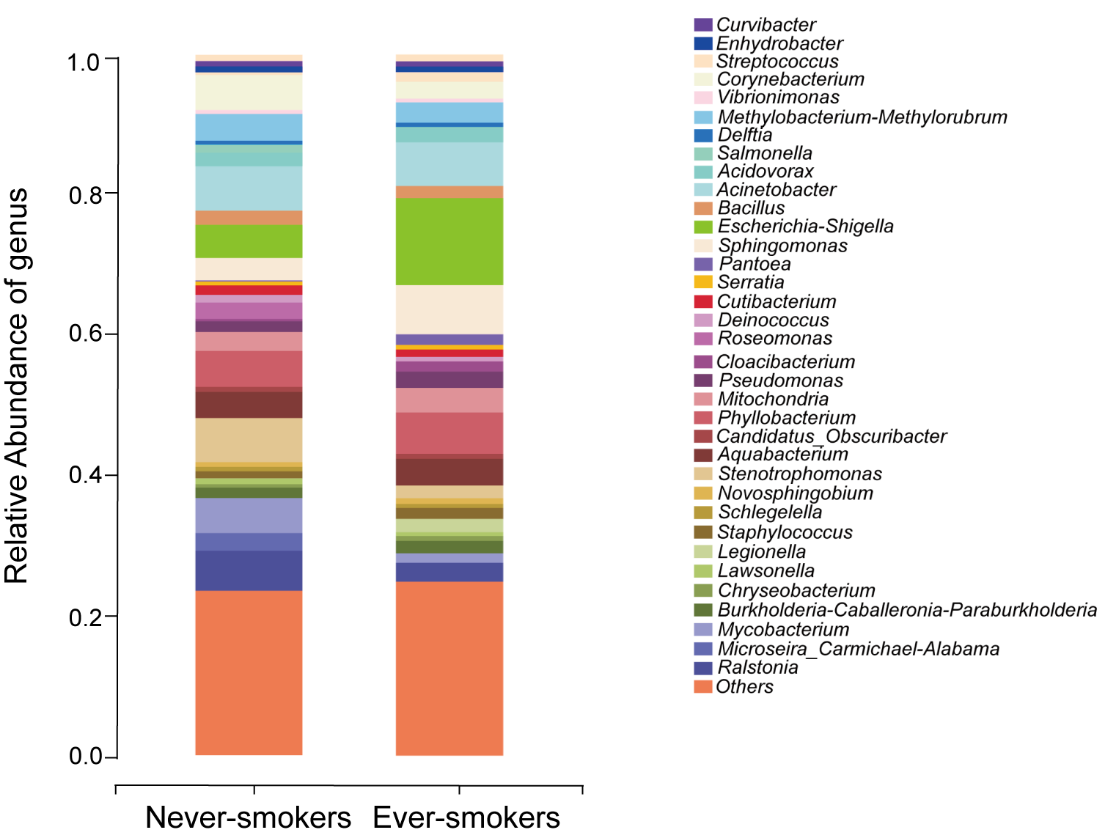


**Supplementary Figure 3.** Relative abundances of the dominant bacterial genus in the microbiota of lung tissues in ever-smoke and never-smoke patients with lung adenocarcinoma


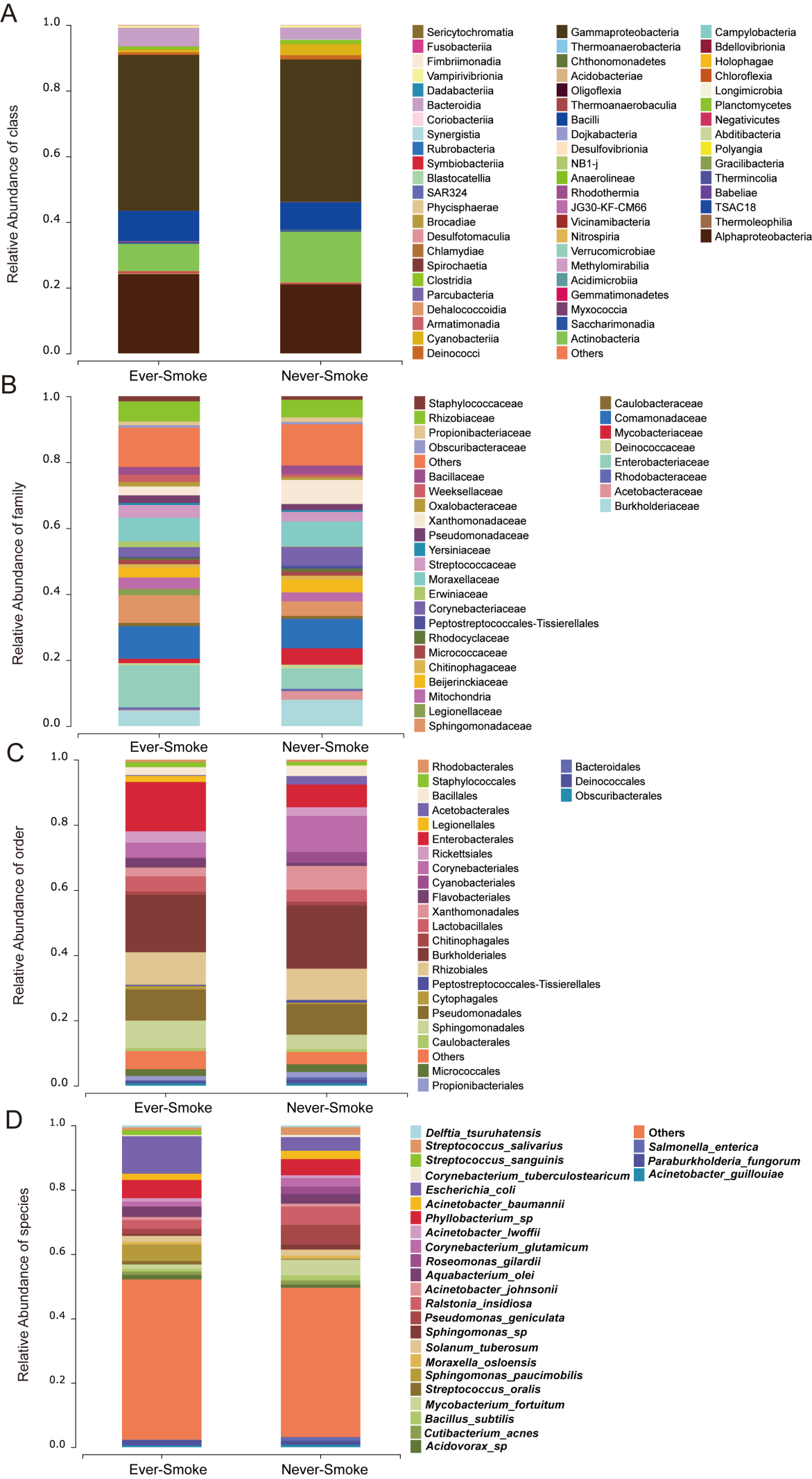


**Supplementary Figure 4.** Relative abundances of the dominant bacterial class **(A)**, family **(B)**, order **(C)**, and species **(D)** in the microbiota of lung tissues in ever-smoke and never-smoke patients with lung adenocarcinoma.


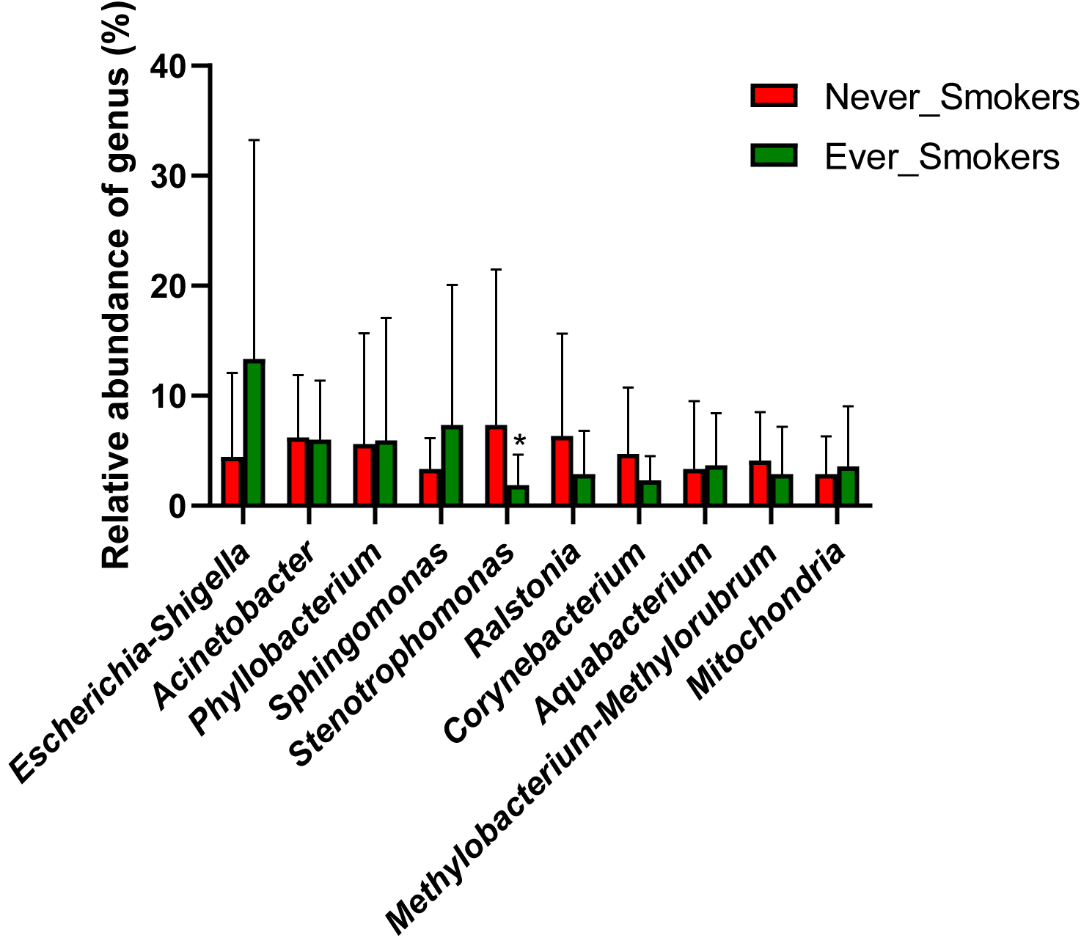


**Supplementary Figure 5.** Relative abundances of the dominant genus in the microbiota of lung tissues in ever-smoke and never-smoke patients with lung adenocarcinoma.


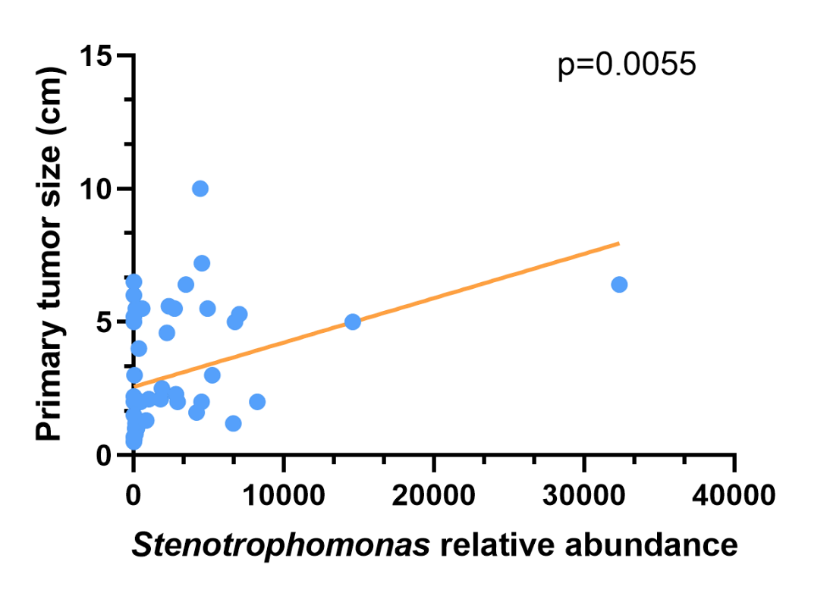


**Supplementary Figure 6.** The relative abundance of *Stenotrophomonas* was significantly positively correlated (p=0.0055) with Primary tumor size of patients with lung adenocarcinoma.


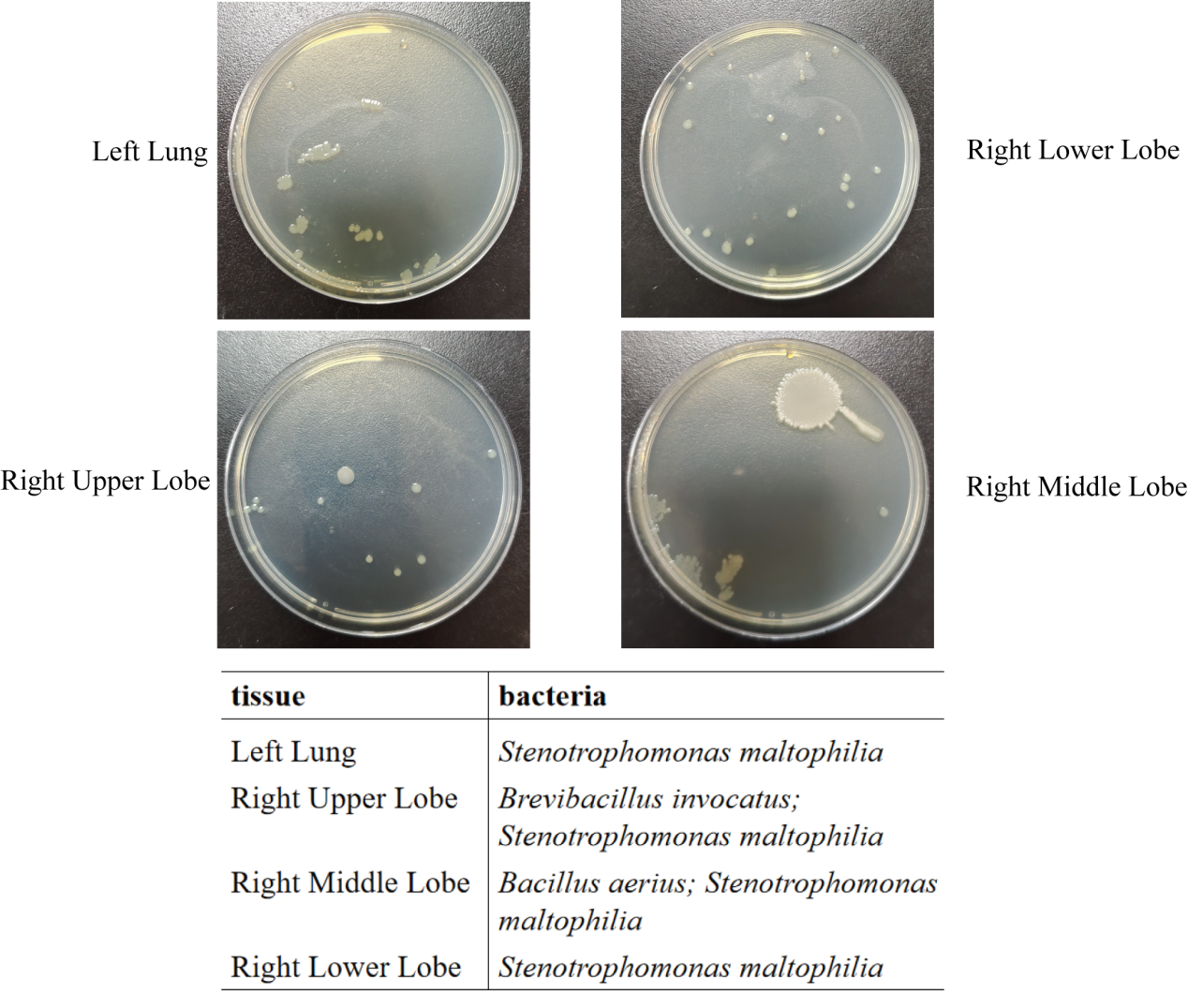


**Supplementary Figure 7.** Representative pictures of bacteria culture analysis of different lung lobes of mice treated with *S. maltophilia*.


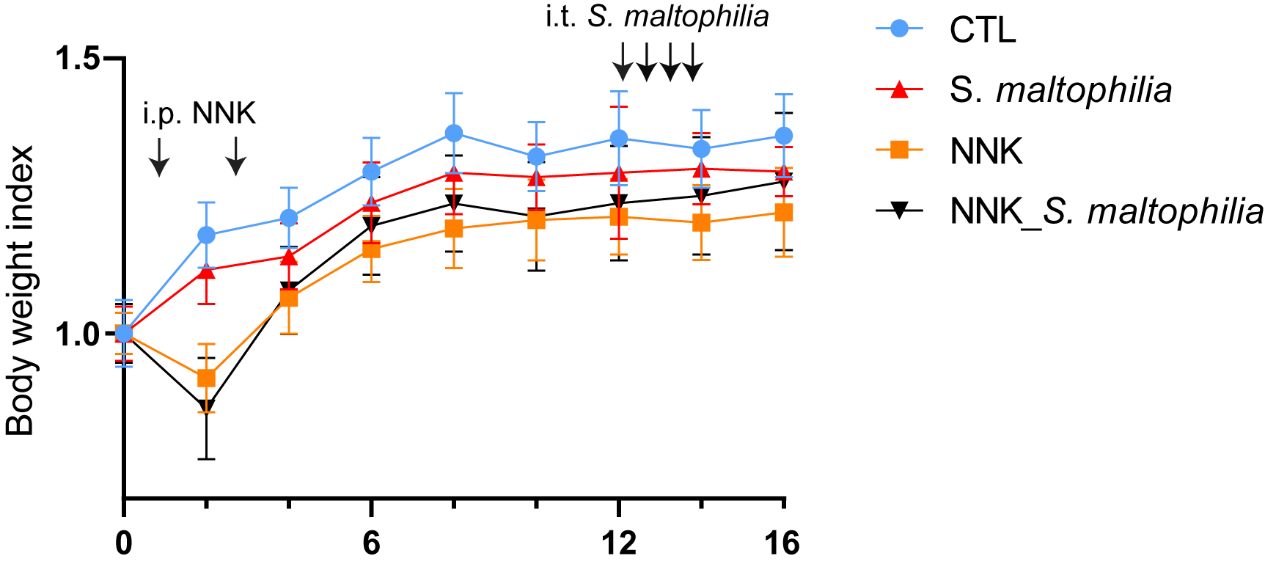


**Supplementary Figure 8.** Weight measurement (the change of weight prior to sacrifice) showing that *S. maltophilia* treatment did not affect the mice's weight gain.


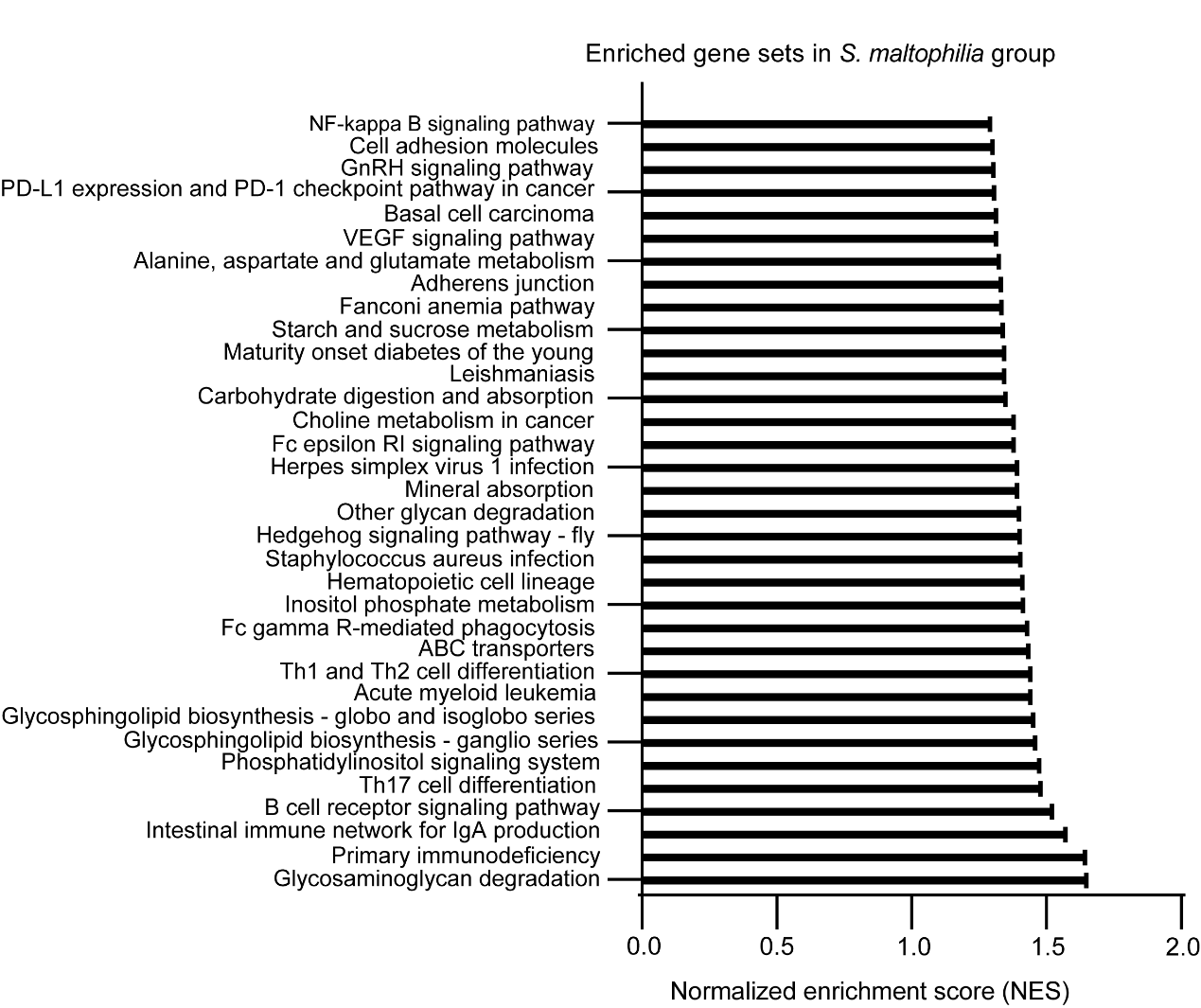


**Supplementary Figure 9.** Normalized Enrichment Score plot of the 34 pathways in *S. maltophilia* group compared with CTL group.


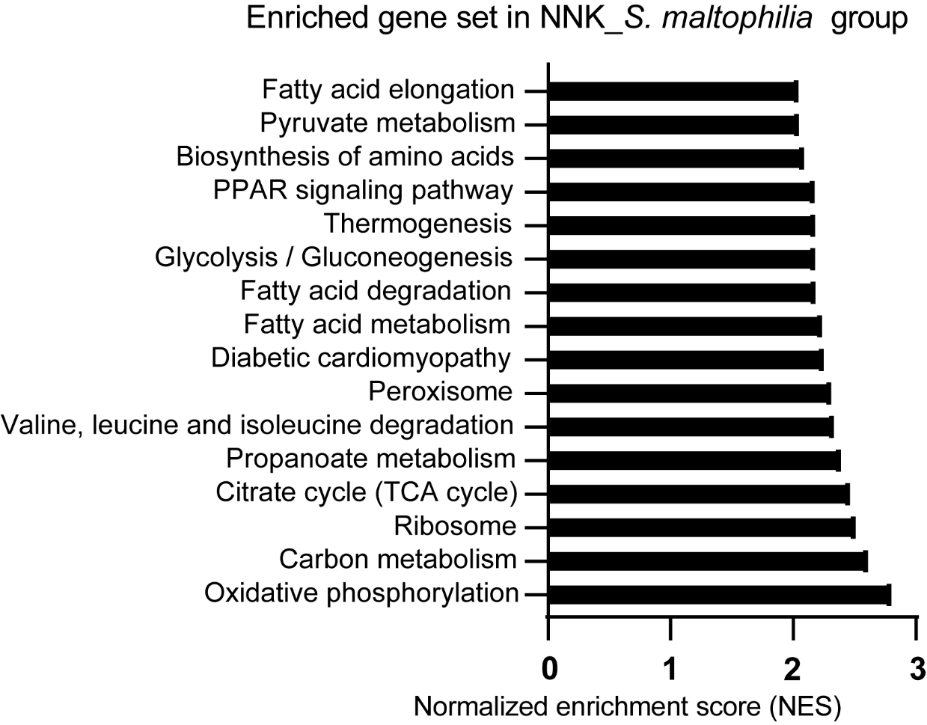


**Supplementary Figure 10.** Normalized Enrichment Score plot of the 16 pathways in NNK_*S. maltophilia* group compared with NNK group.


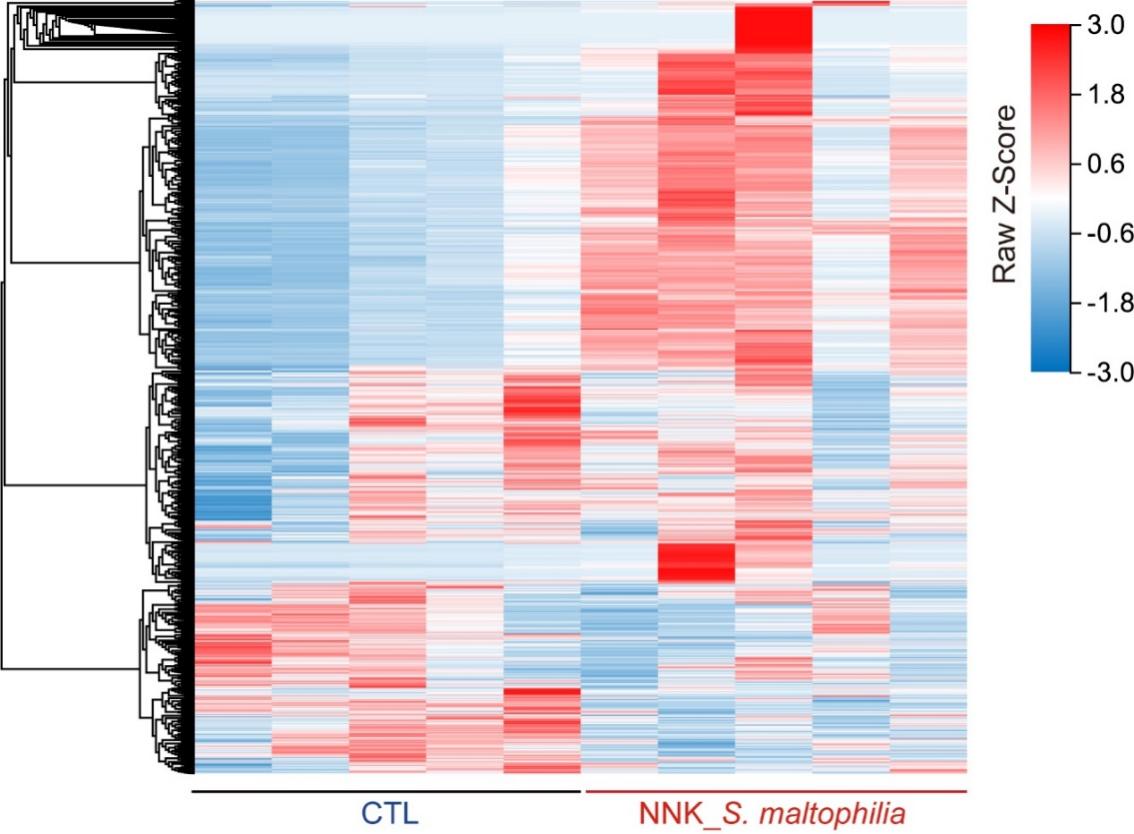


**Supplementary Figure 11.** Heatmap of different genes in lung tissues of NNK_*S. maltophilia* group compared with CTL group.


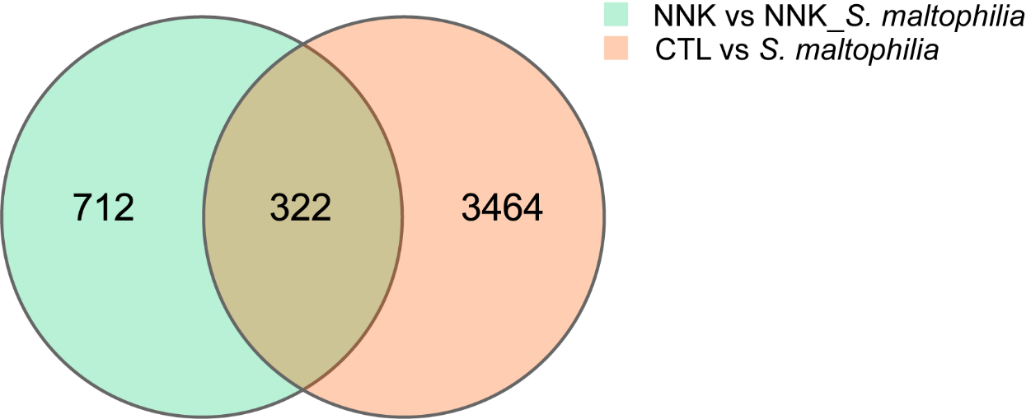


**Supplementary Figure 12.** Venn diagram of DEGs between the CTL-vs-*S. maltophilia*, and NNK-vs-NNK_*S. maltophilia*.


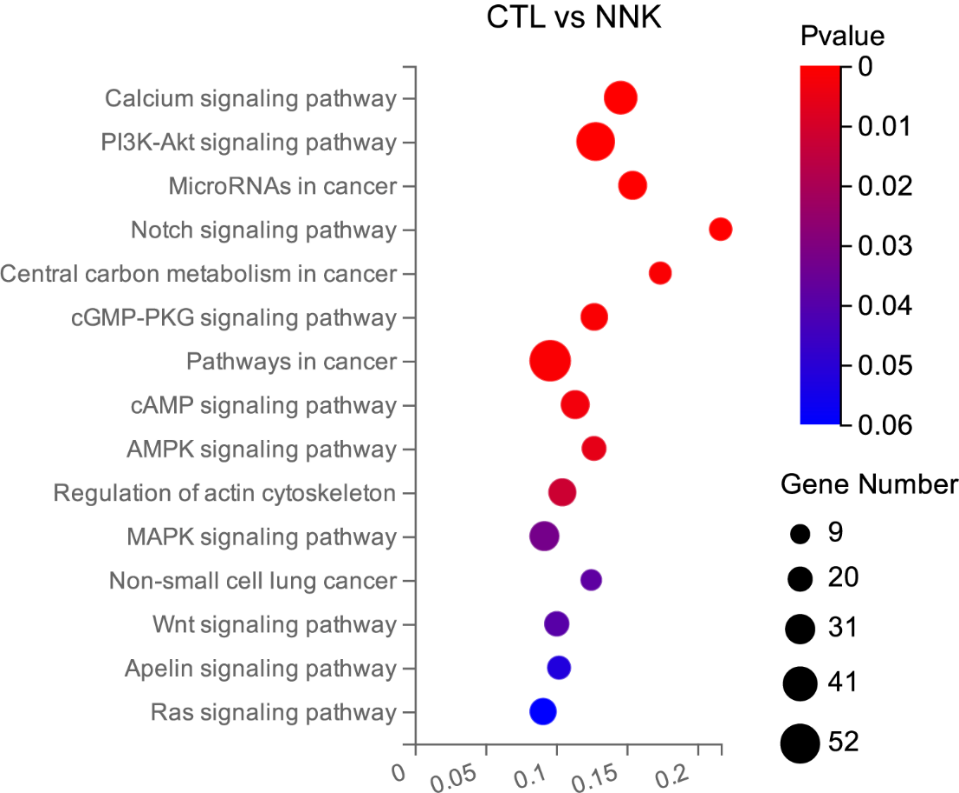


**Supplementary Figure 13.** KEGG pathway enrichment analysis of upregulated genes of RNA-seq data between CTL and NNK groups.
