# Supplementary figures and images for: Stenotrophomonas maltophilia promotes lung adenocarcinoma progression by upregulating histone deacetylase 5
Source: Front Microbiol. 2023 Feb 1;14:1121863. doi: 10.3389/fmicb.2023.1121863 (PMC9929947; doi:10.3389/fmicb.2023.1121863)

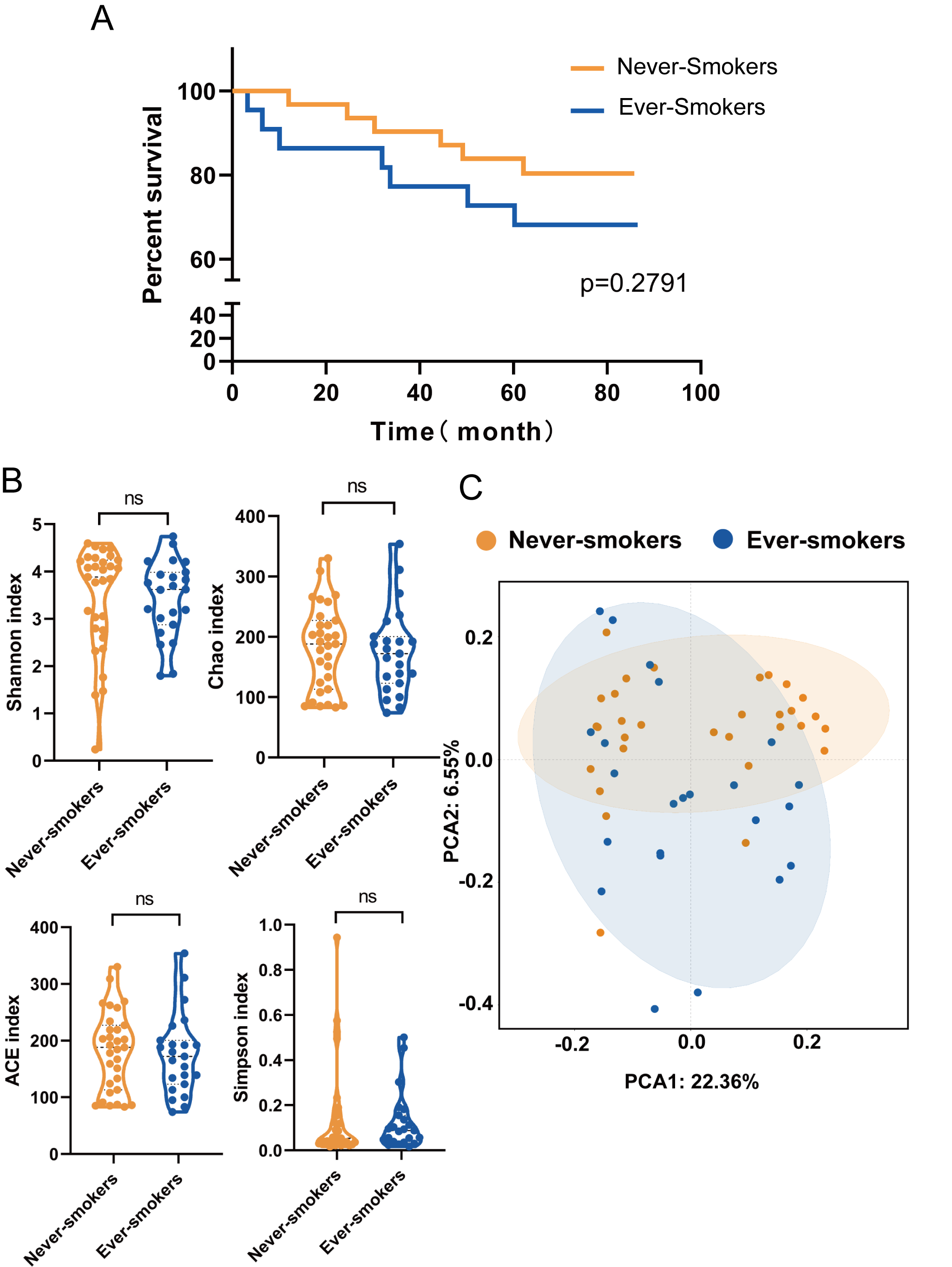

Supplement: Supplementary file 1 [file Data_Sheet_1.ZIP › Supplements/Figure S1.tif]

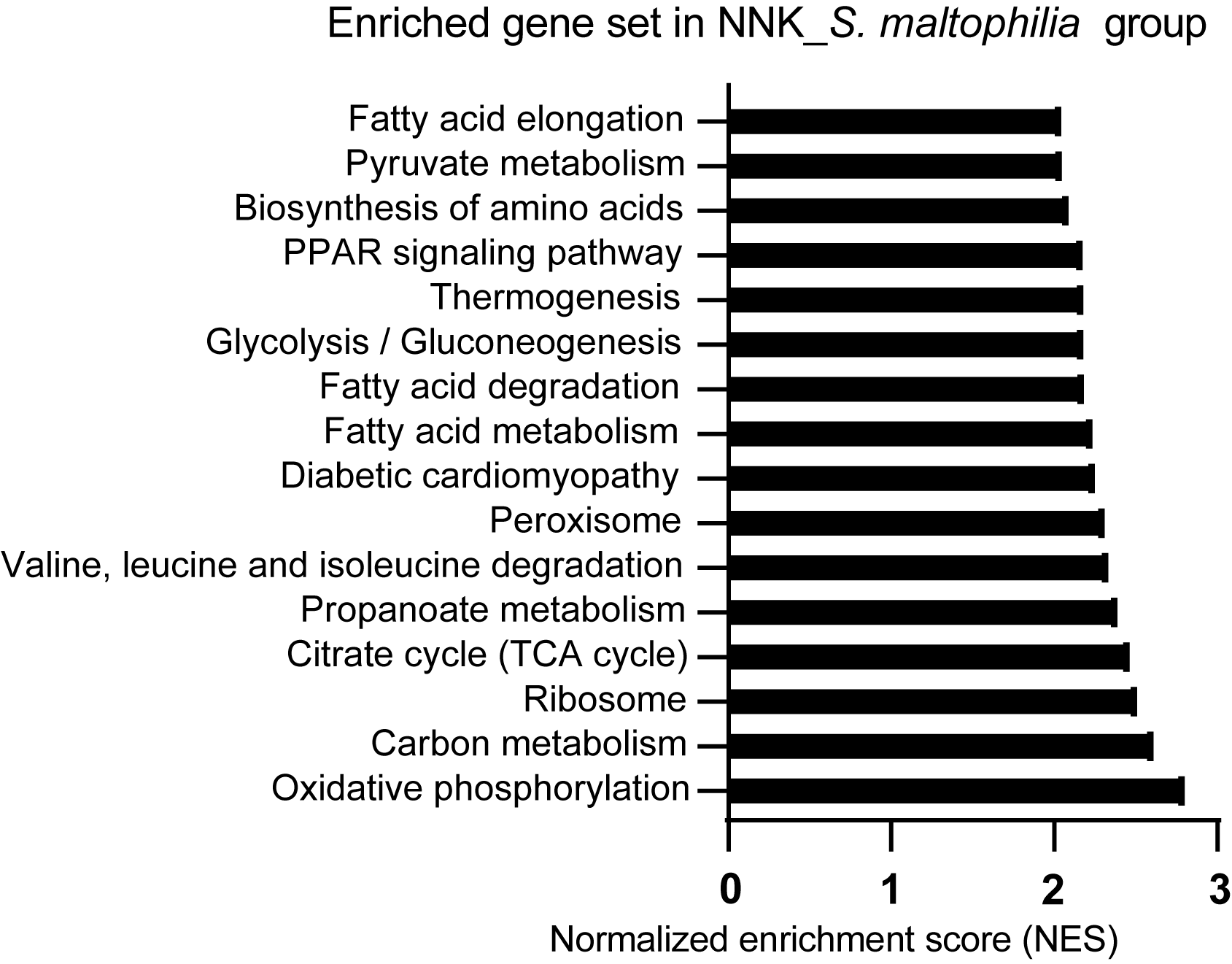

Supplement: Supplementary file 1 [file Data_Sheet_1.ZIP › Supplements/Figure S10 GSEA BAS-NNK╗∙╥≥╕╗╝»═╝.tif]

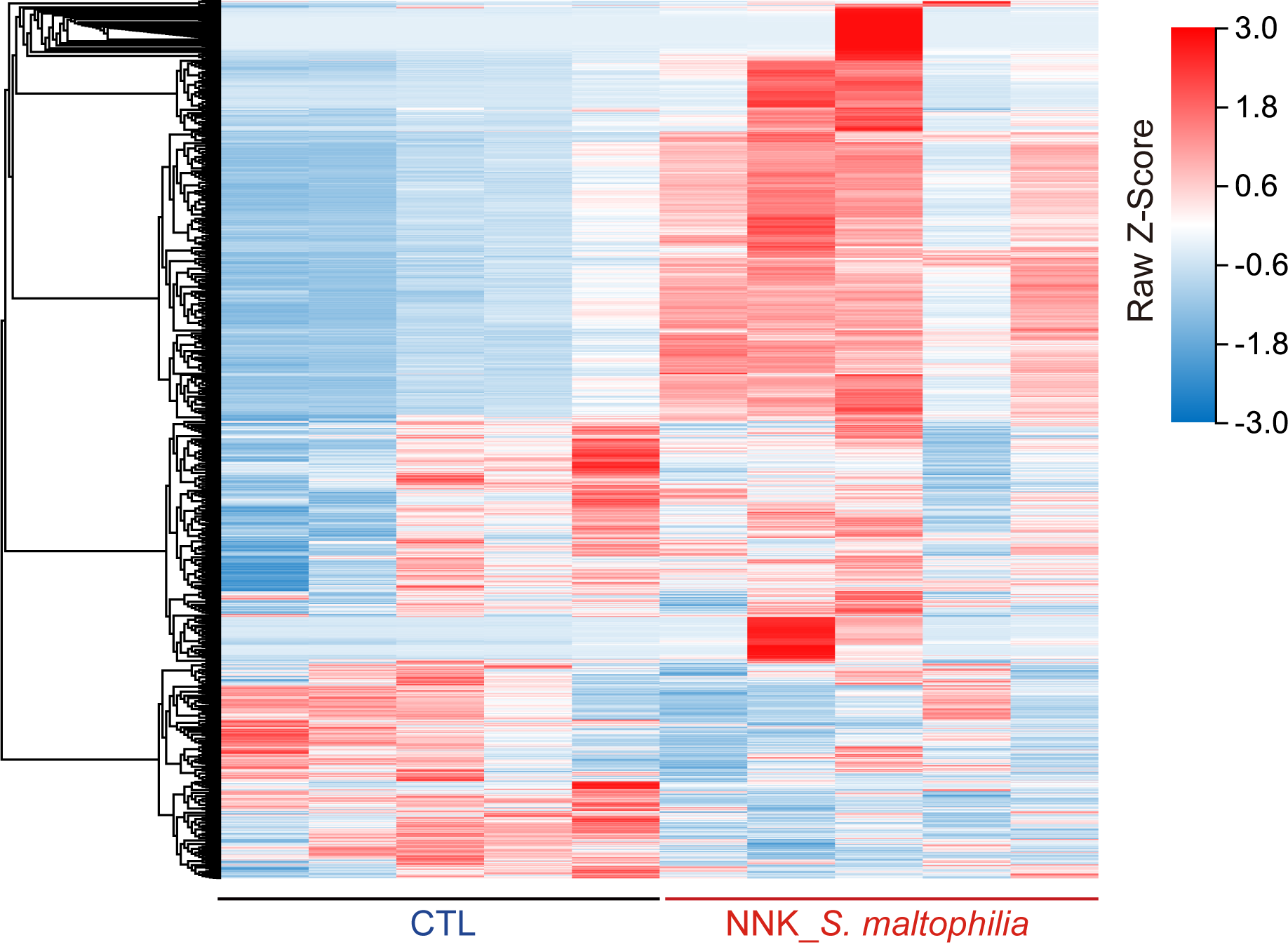

Supplement: Supplementary file 1 [file Data_Sheet_1.ZIP › Supplements/Figure S11 (2).tif]

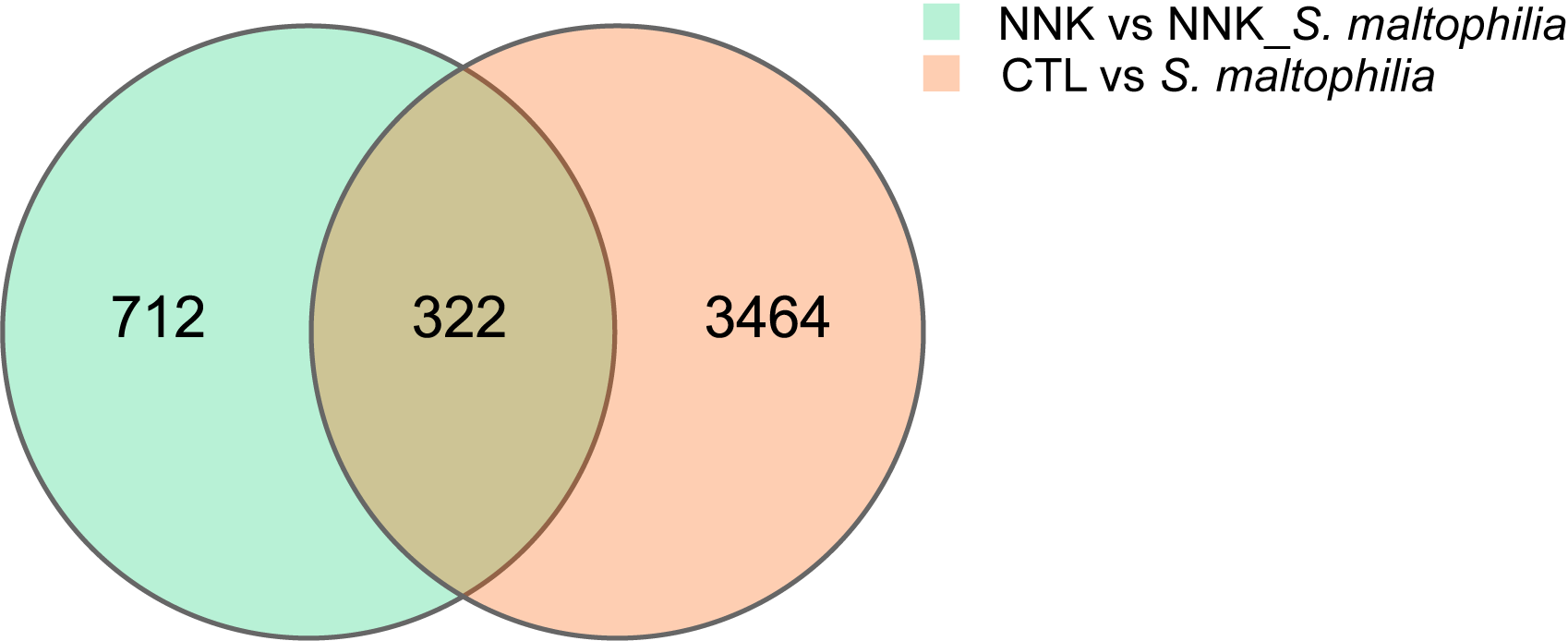

Supplement: Supplementary file 1 [file Data_Sheet_1.ZIP › Supplements/Figure S12.tif]

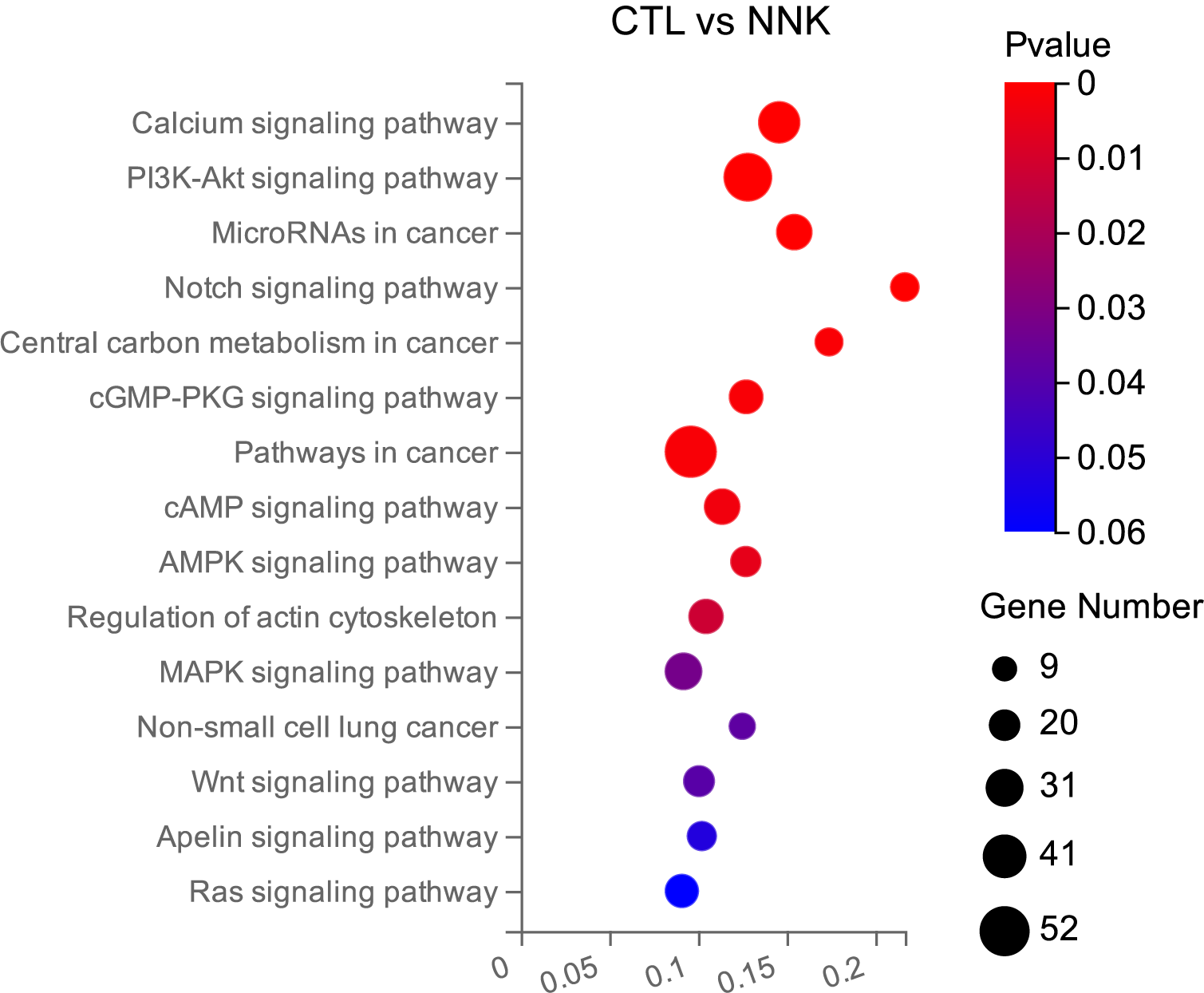

Supplement: Supplementary file 1 [file Data_Sheet_1.ZIP › Supplements/Figure S13.tif]

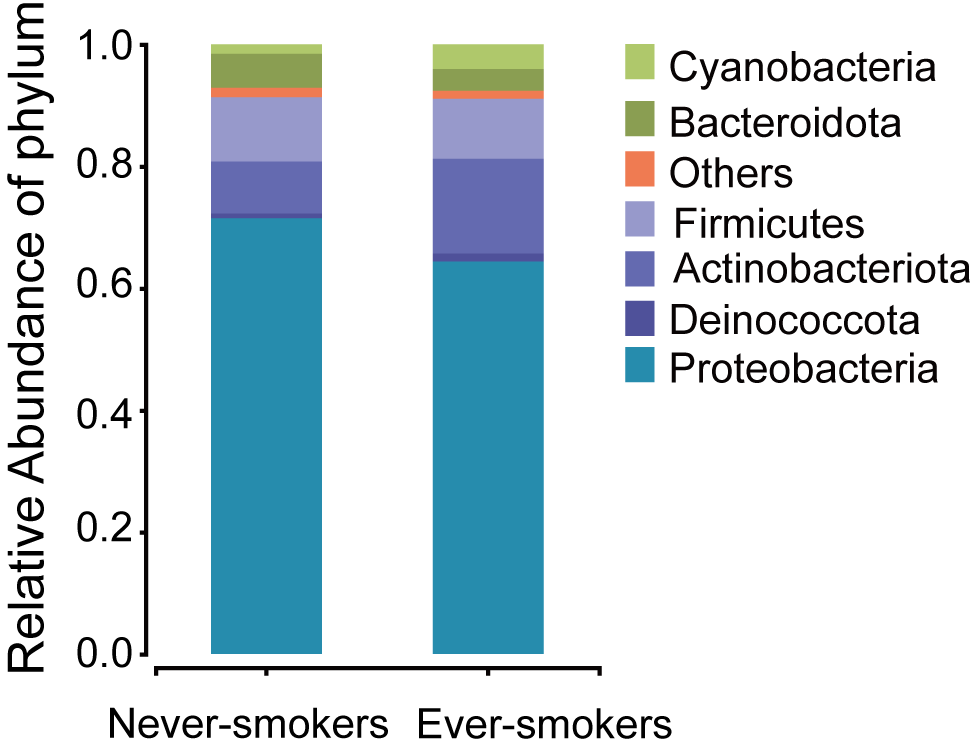

Supplement: Supplementary file 1 [file Data_Sheet_1.ZIP › Supplements/Figure S2e.tif]

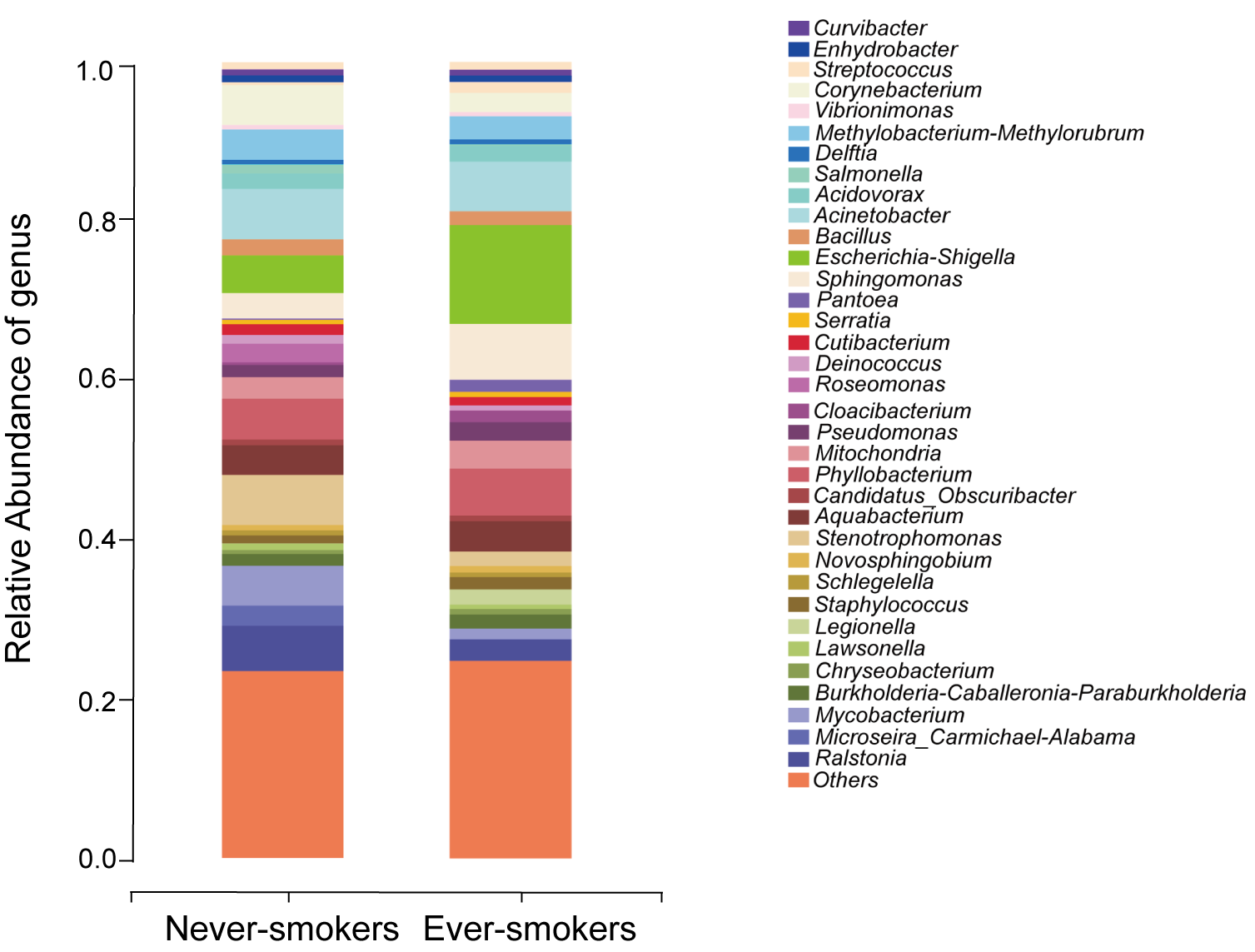

Supplement: Supplementary file 1 [file Data_Sheet_1.ZIP › Supplements/Figure S3e.tif]

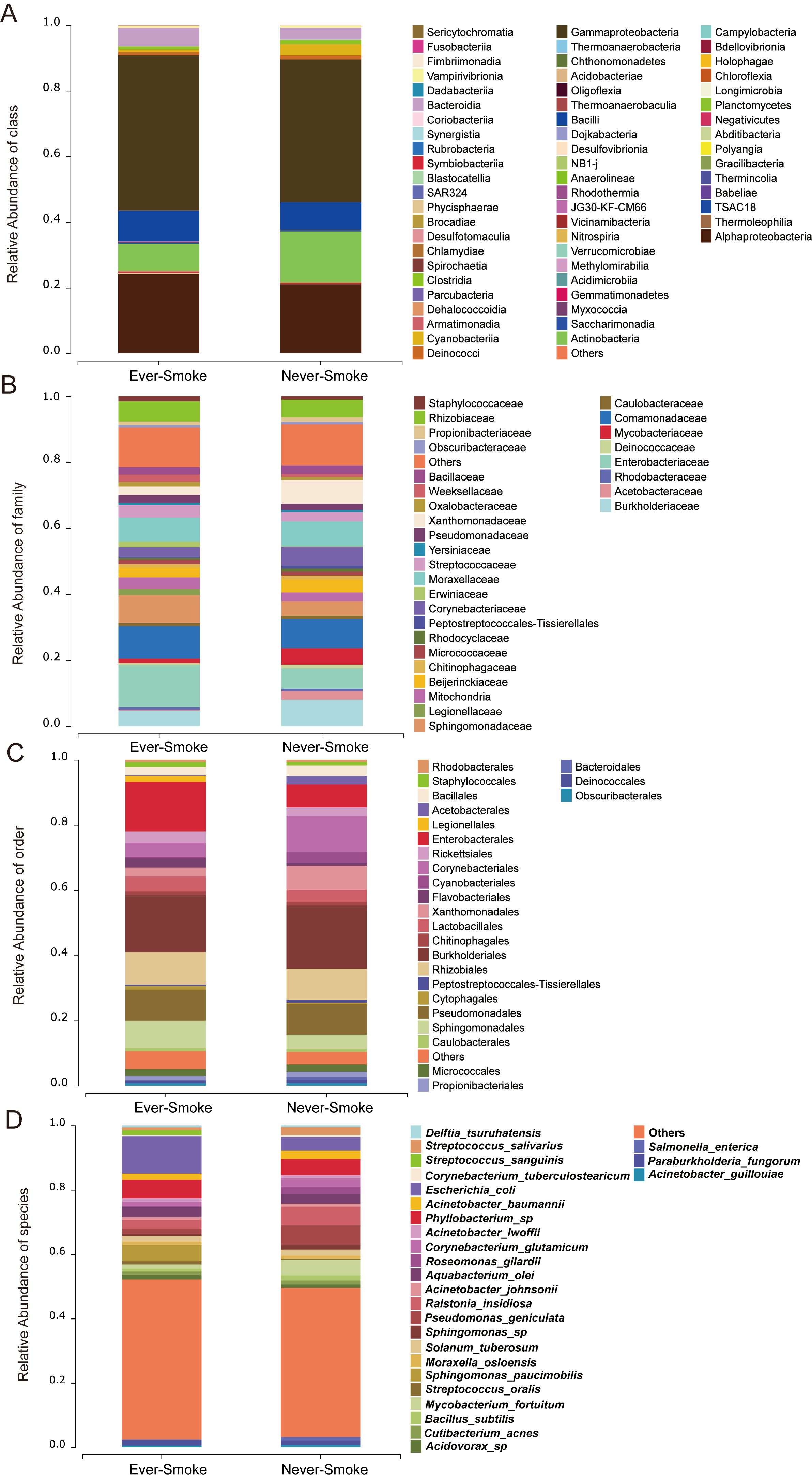

Supplement: Supplementary file 1 [file Data_Sheet_1.ZIP › Supplements/Figure S4.tif]

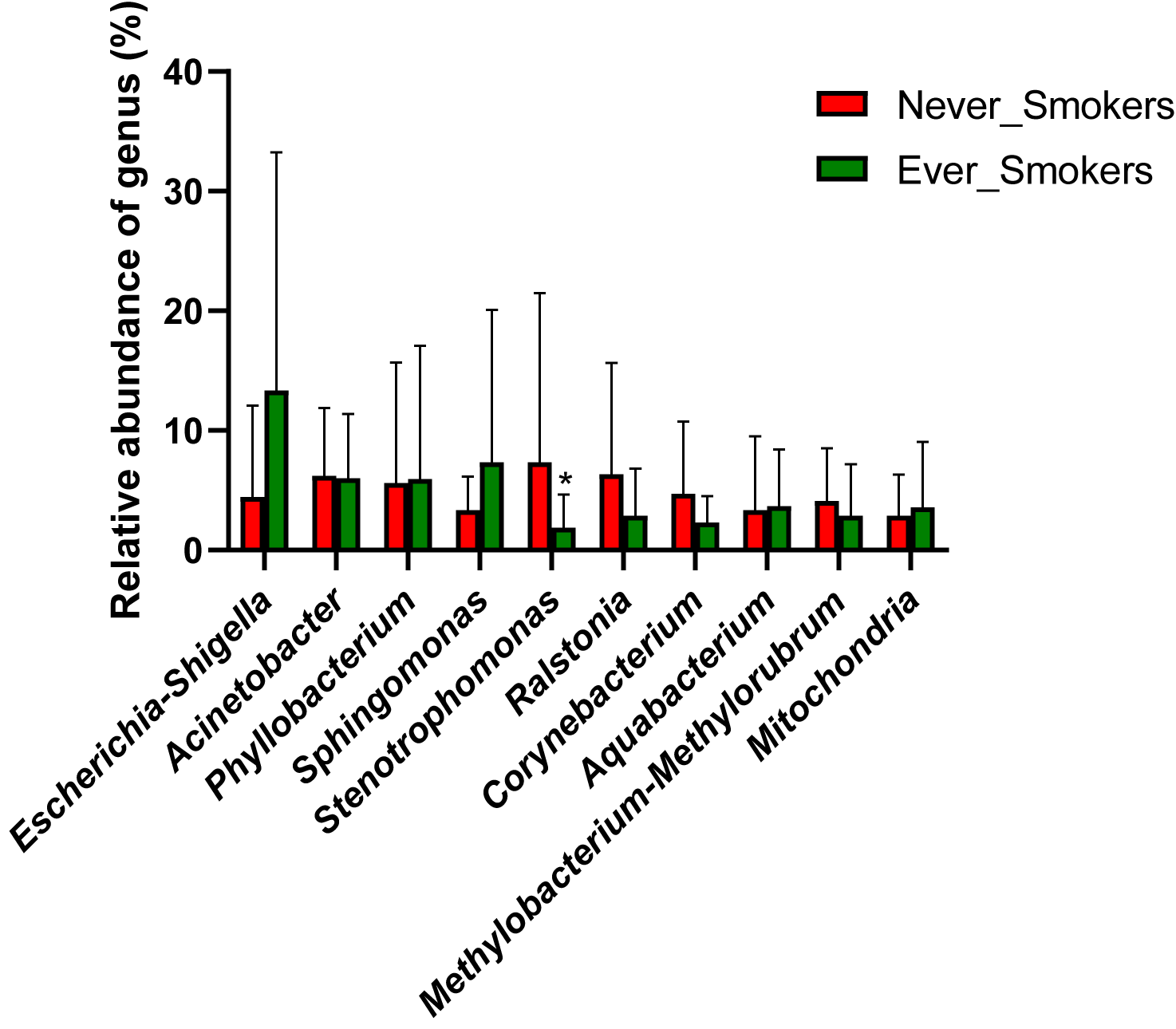

Supplement: Supplementary file 1 [file Data_Sheet_1.ZIP › Supplements/Figure S5e.tif]

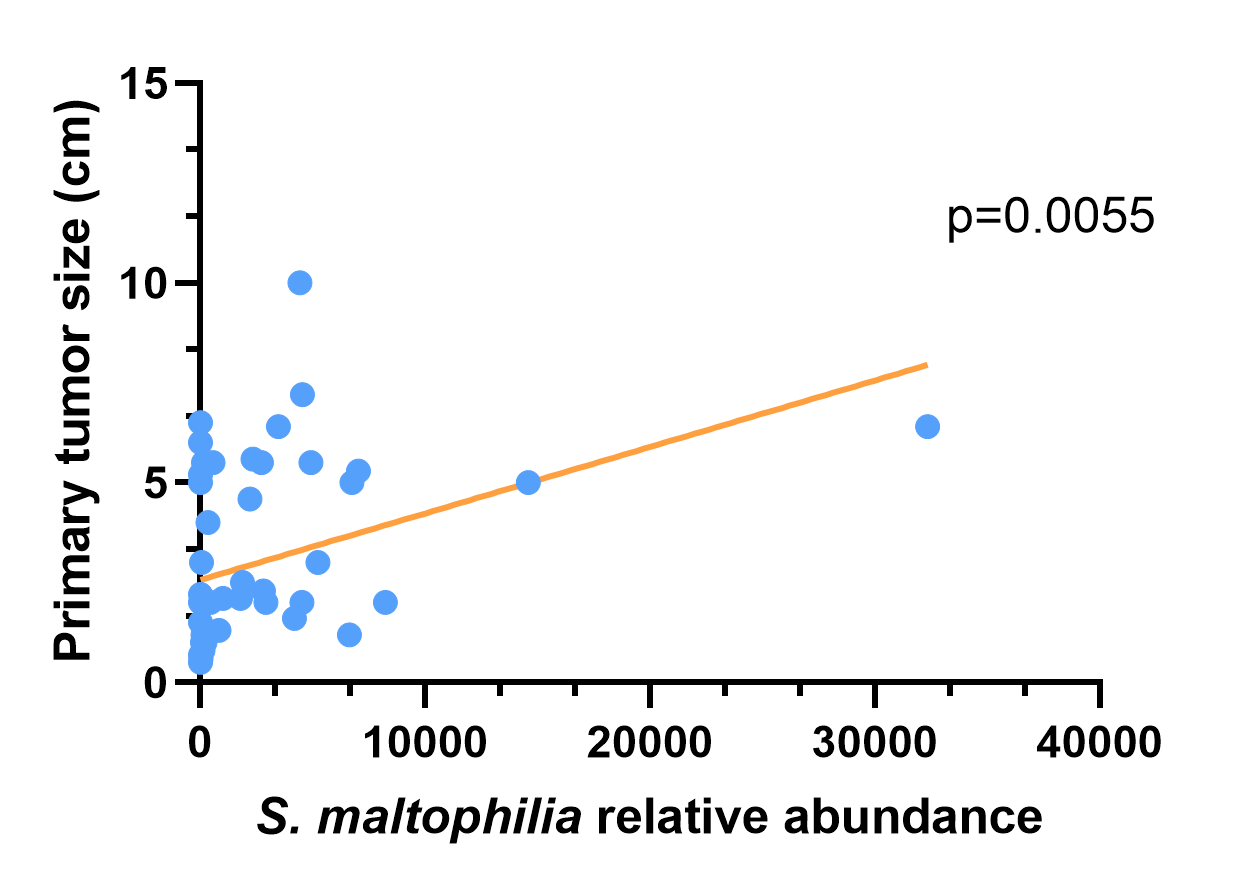

Supplement: Supplementary file 1 [file Data_Sheet_1.ZIP › Supplements/Figure S6e.tif]

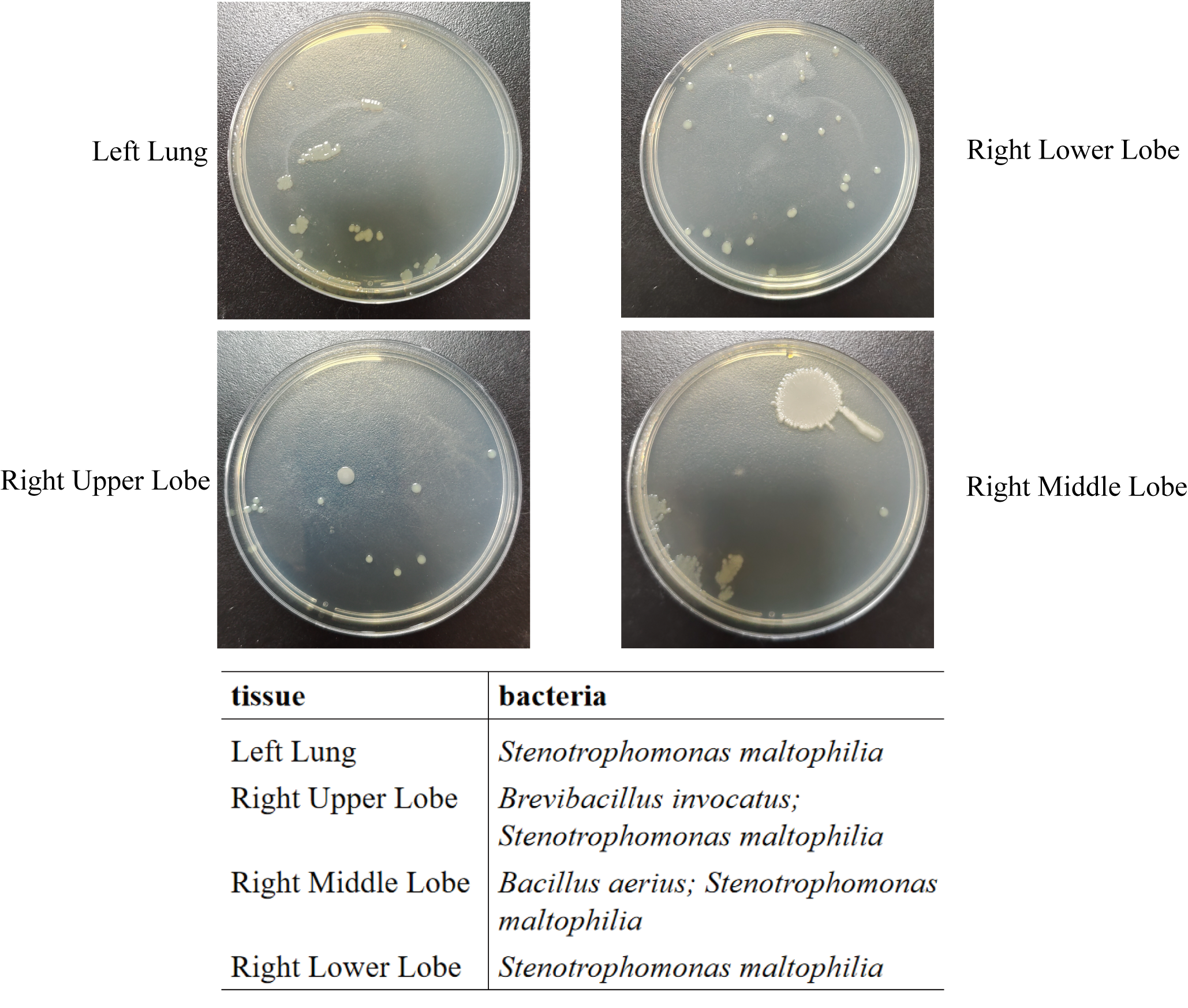

Supplement: Supplementary file 1 [file Data_Sheet_1.ZIP › Supplements/Figure S7.tif]

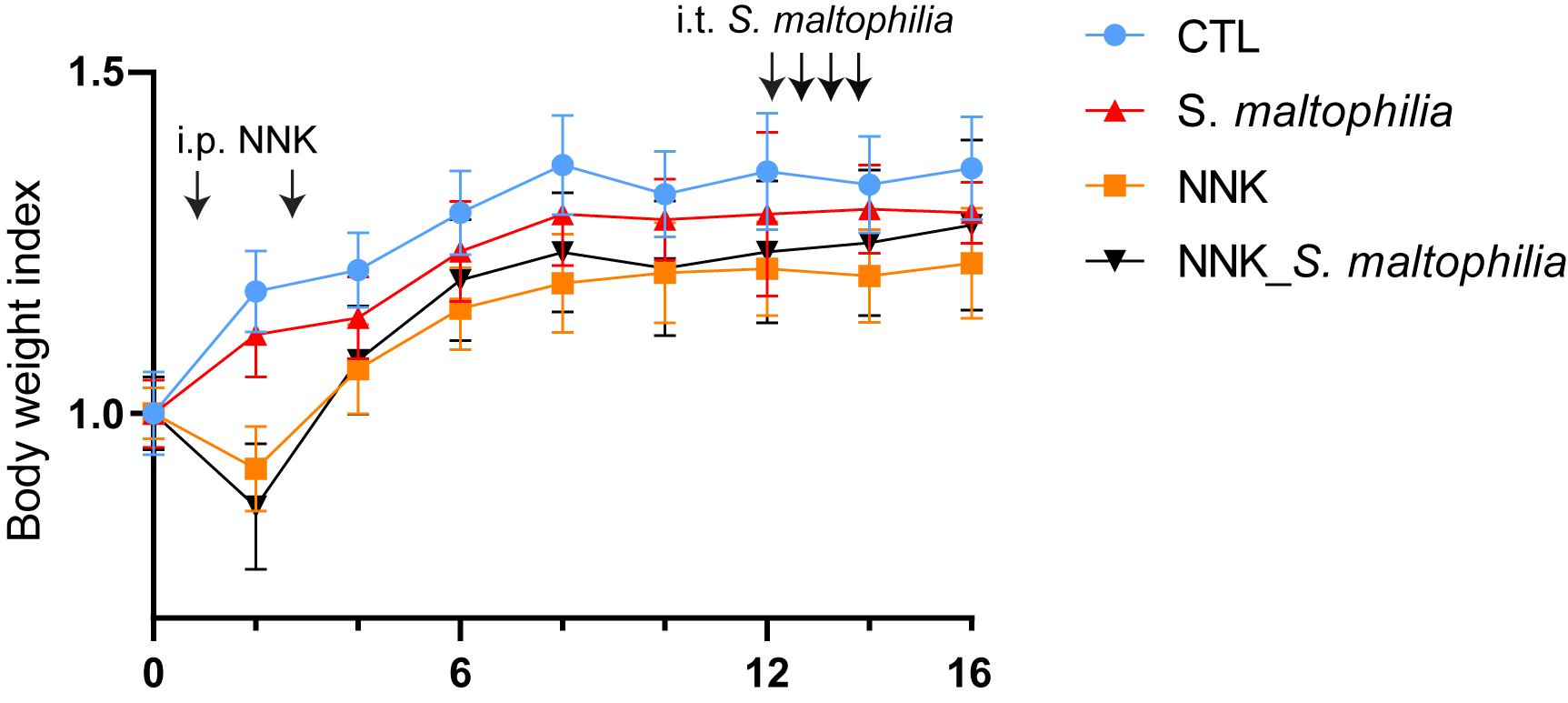

Supplement: Supplementary file 1 [file Data_Sheet_1.ZIP › Supplements/Figure S8.tif]

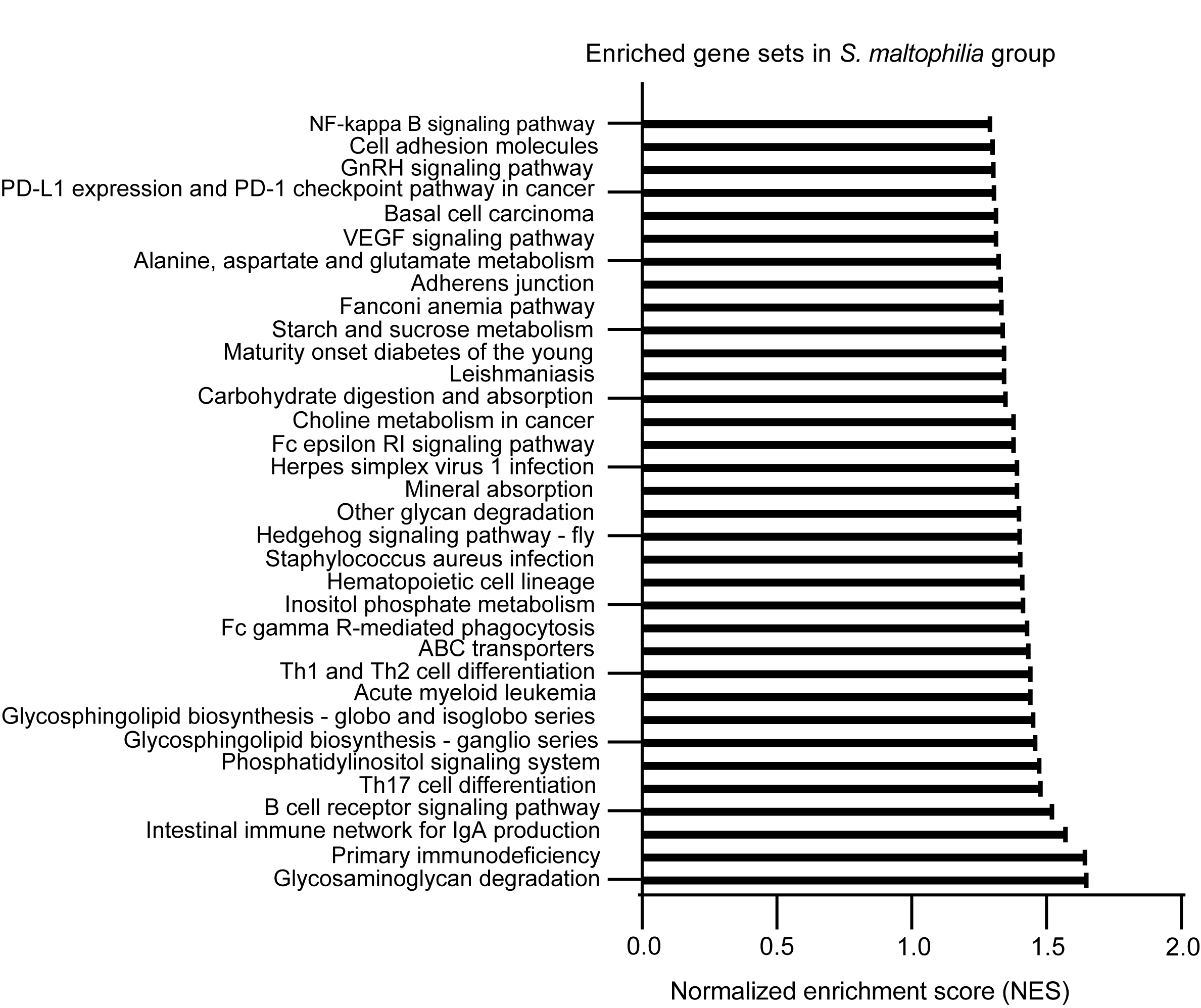

Supplement: Supplementary file 1 [file Data_Sheet_1.ZIP › Supplements/Figure S9 GFSE BAS╫Θ╕╗╝»╗∙╥≥ú¿34)+.tif]
